# Speleothem growth intervals reflect New Zealand montane vegetation response to temperature change over the last glacial cycle

**DOI:** 10.1038/s41598-020-58317-8

**Published:** 2020-02-12

**Authors:** John Hellstrom, Kale Sniderman, Russell Drysdale, Isabelle Couchoud, Adam Hartland, Andrew Pearson, Petra Bajo

**Affiliations:** 1 School of Earth Sciences, University of Melbourne, Victoria, 3010 Australia; 2 School of Geography, University of Melbourne, Victoria, 3010 Australia; 3 Laboratoire EDYTEM, CNRS, Université Savoie Mont Blanc, Université Grenoble Alpes, Chambéry, France; 4 Environmental Research Institute, School of Science, University of Waikato, Private Bag 3105, Hamilton, 3240 New Zealand; 5 Croatian Geological Survey, Sachsova 2, 10 000 Zagreb, Croatia

**Keywords:** Palaeoclimate, Geochemistry

## Abstract

Flowstone speleothem growth beneath Mount Arthur, New Zealand shows a clear relationship to vegetation density and soil development on the surface above. Flowstone does not currently form beneath sub-alpine *Nothofagus* forest above ca. 1000–1100 m altitude but U-Th dating shows it has formed there during past intervals of warmer-than-present conditions including an early–mid Holocene optimum and the last interglacial from ca. 131–119 ka. Some flowstones growing beneath ca. 600 m surface altitude, currently mantled with dense broadleaf-podocarp forest, grew during full glacial conditions, indicating that local tree line was never below this altitude. This implies that Last Glacial Maximum annual temperature was no more than ca. 4 °C cooler than today. Flowstone growth appears to be a robust indicator of dense surface vegetation and well-developed soil cover in this setting, and indicates that past interglacial climates of MIS 7e, 5e, the early–mid Holocene and possibly MIS 5a were more conducive to growth of trees than was the late Holocene, reflecting regional temperature changes similar in timing to Antarctic temperature changes. Here, flowstone speleothem growth is a sensitive indicator of vegetation density at high altitude, but may respond to other factors at lower altitudes.

## Introduction

Speleothems are secondary carbonate precipitates, usually calcite, which form in caves. To grow, they require elevated CO_2_ in the soil atmosphere above them, enough effective precipitation to dissolve and transport that CO_2_, a flow path to a cave allowing sufficient water-rock interaction with soluble minerals to saturate with carbonate, and long enough residence time in a relatively low-CO_2_ cave atmosphere to supersaturate and precipitate calcite^1,2^. Speleothems have often been used to reconstruct past climates and environments, usually by interpreting their records of stable isotope or other geochemical variation over time, dated using U–Th^3^. An alternative approach to reconstructing past climates has been to use variability in speleothem growth rate and frequency to infer past changes in environmental conditions that favour speleothem growth^4–6^. Intermittent and/or variable speleothem growth has been interpreted to indicate changes in precipitation^5^, temperature^7^, ice cover^8^, cave ventilation^9^ and vegetation density^7,10,11^, depending on which of these are thought to be the limiting factor in a given setting. Most speleothem paleoenvironmental records are derived from stalagmites^3^ but flowstones, sheet speleothems formed on walls and floors, have also been used^12–14^. Flowstones are often relatively small but sometimes more than a metre thick and tens of square metres in extent.

**Figure 1:**
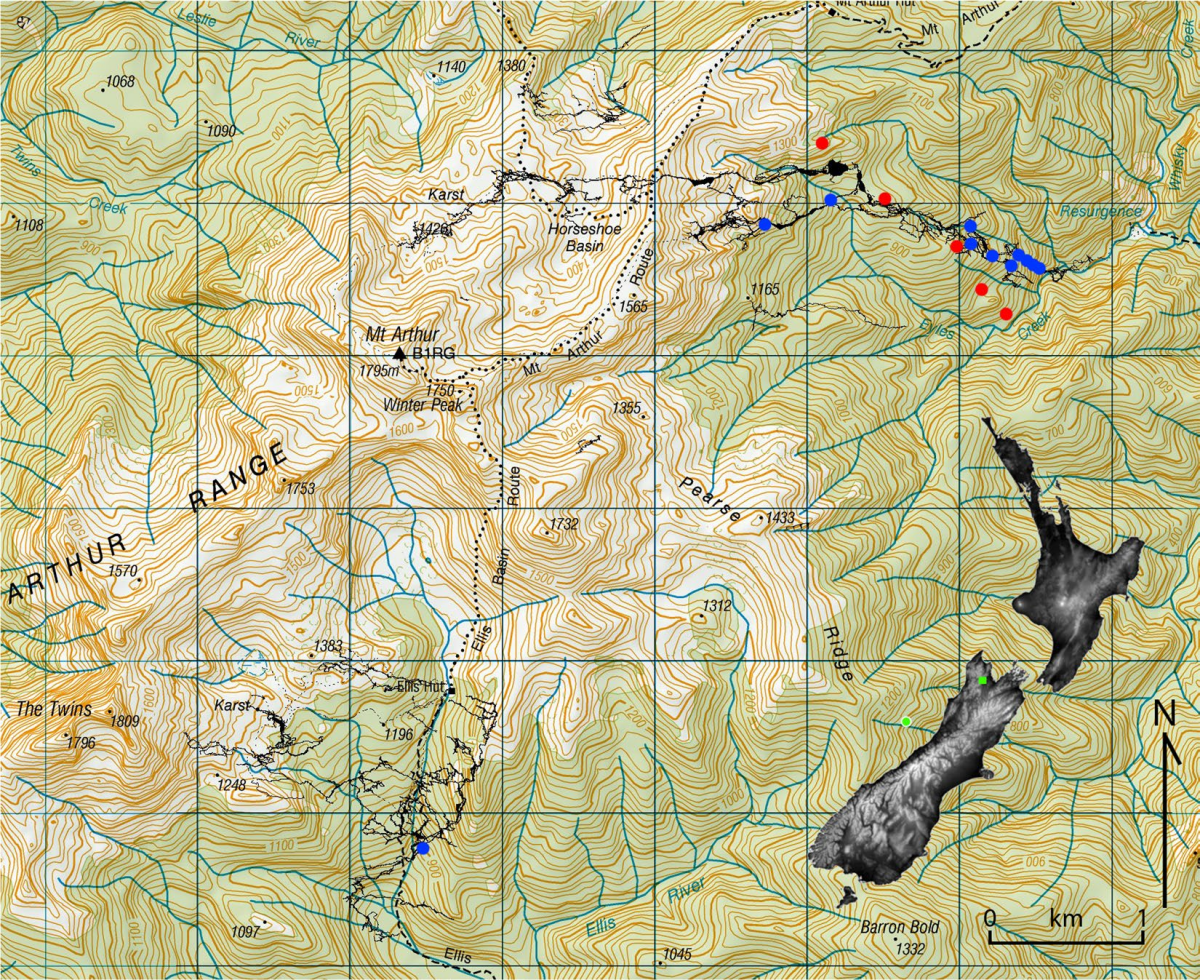
Topographical map of Mount Arthur showing cave outlines (filled black sinuous lines), locations of flowstone core samples (blue) and locations of soil pits (red). Three flowstones and one soil pit are located ca. 2 km north of this map at ca. 950 m asl (exact location cannot be shown). Contour interval is 20 m. Inset map is shaded by altitude; green box shows location of Mount Arthur and green circle shows relative location of marine cores TAN0513-14^45^ and SO136-GC3^44^. Cave outlines were provided by Jonathan Ravens. Base map source: Land Information New Zealand (LINZ), licensed by LINZ for re-use under the Creative Commons Attribution 4.0 International licence https://www.linz.govt.nz/land/maps. Inset map source: GTOPO30 elevation data courtesy of the U.S. Geological Survey doi: 10.5066/F7DF6PQS.

Mount Arthur rises to 1803 m in the north-west corner of New Zealand’s South Island, at latitude 41.5° south (Fig. 1), entirely within Kahurangi National Park beneath undisturbed native vegetation. It is predominantly composed of Ordovician marble of up to 2000 m thickness and is underlain by many known caves, the two largest of which are almost 40 km in length and over 1000 m in depth. Demonstrated hydrological connections suggest that the individual cave systems are linked^15^, implying hundreds of km of cave passage remain to be discovered there. A smaller area of Oligocene limestone, less than 100 m thick, overlies the northern part of the mountain and contains a number of much smaller known caves. Speleothems from Mount Arthur have been shown to be sensitive to external climatic variability^14^ and, using magnetostratigraphic dating, have previously been found to grow over at least the last 775,000 years^16,17^. The current tree line, corresponding to a coldest month mean temperature of about 0 °C^18^ is 1200–1300 m above sea level (asl), above which there are alpine grass and herb fields with bare marble in the most exposed areas and limited areas of woody shrubland in the most sheltered areas. Current mean annual temperature (MAT) ranges from ca. 10 °C at the foot of the mountain (270 m asl) to less than 5 °C at its summit^19^. Current mean annual precipitation has a strong relationship to altitude, being greater than 2500 mm on the upper slopes of the mountain (above ca. 1500 m asl), reducing to ca. 1500 mm at the floor of the Motueka River valley (ca. 75 m asl and 10 km to the east)^19^.

Early work at Mount Arthur suggested that speleothem growth rate was controlled by vegetation density^7,14^, and the importance of vegetation density has subsequently been indicated in other settings. For example the commencement of stalagmite growth in an abandoned mine in Wiltshire, England, was a response to enhanced soil pCO_2_ driven by regrowth of forest vegetation above the mine, some decades after abandonment^10^. Likewise, montane Italian speleothems exhibit a growth rate response to tree line altitude, including to higher-than-current altitudes during the last interglacial^11^. Here, we systematically explore the question of controls of vegetation density on speleothem growth and their relationship to changing climate in a cool temperate montane setting, inspired by the observation that flowstones are common beneath forested surfaces of the Mount Arthur, but very rare beneath its alpine terrain. Suitable flowstones were located in Nettlebed Cave beneath the south-eastern slopes of the mountain, underlying surface altitudes between 500 m asl (where they are ca. 50 m above the current active phreatic zone of the cave) and 1200 m asl, the current treeline above the cave. One additional flowstone was sampled from ExhaleAir Cave beneath the southern slopes, and three flowstones were sampled from a shallow limestone cave on the north-western slopes of the mountain. In this paper, we discuss intervals of flowstone growth at Mount Arthur over the last glacial cycle in terms of past New Zealand climate^20^.

## Materials and Methods

### Sample recovery

Twenty flowstones were selected from areas within Mount Arthur caves which are overlain by terrain ranging in surface altitude from 500 to 1200 metres asl. Flowstones were sampled by coring, with speleothems selected for thickness and relatively laminar growth layering, to the extent that it is possible to judge these from external morphology. Fifteen of these speleothems gave core samples of at least ca. 100 mm of relatively clean and void-free calcite (Table 1), predominantly of columnar fabric^2^ (Supplementary figure 1). Cores MD3 and ED1, 50 mm diameter, were drilled using a custom-built petrol-powered drilling system with exhaust capture and purification. Cores NB3, NB5 and NB7 to NB9, 22 mm diameter, were collected using a Pomeroy Industries DE-T3 NiMH-powered electric drilling system. Cores NB11, NB15-x and HC15-x, 48 mm diameter, were collected using a custom Pomeroy Industries Li-Ion-powered electric drilling system. Core recovery was largely continuous, with individual segment lengths of between ca. 10 mm and 400 mm, labelled for orientation and sequence during collection.

**Table 1:** Mount Arthur speleothem core samples arranged by altitude of the overlying surface. Length is of total recovered core sample and basal age is the deepest U-Th age measurement successfully obtained.

Core	surface altitude (m)	length (mm)	Basal age (ka)	Number of U-Th ages
NB11	490	452	121	64
NB3	500	358	162	35
NB15-3	540	376	70	10
NB15-2	560	294	66	9
MD3	590	595	32	60
NB5	650	496	95	12
NB15-1	680	1011	129	13
NB6	760	196	120	5
NB7	785	277	117	7
ED1	870	233	84	15
HC15-2	940	89	14	9
HC15-1	945	290	35	7
HC15-3	950	450	87	10
NB9	1010	143	> 550	15
NB8	1210	499	248	18

**Figure 2:**
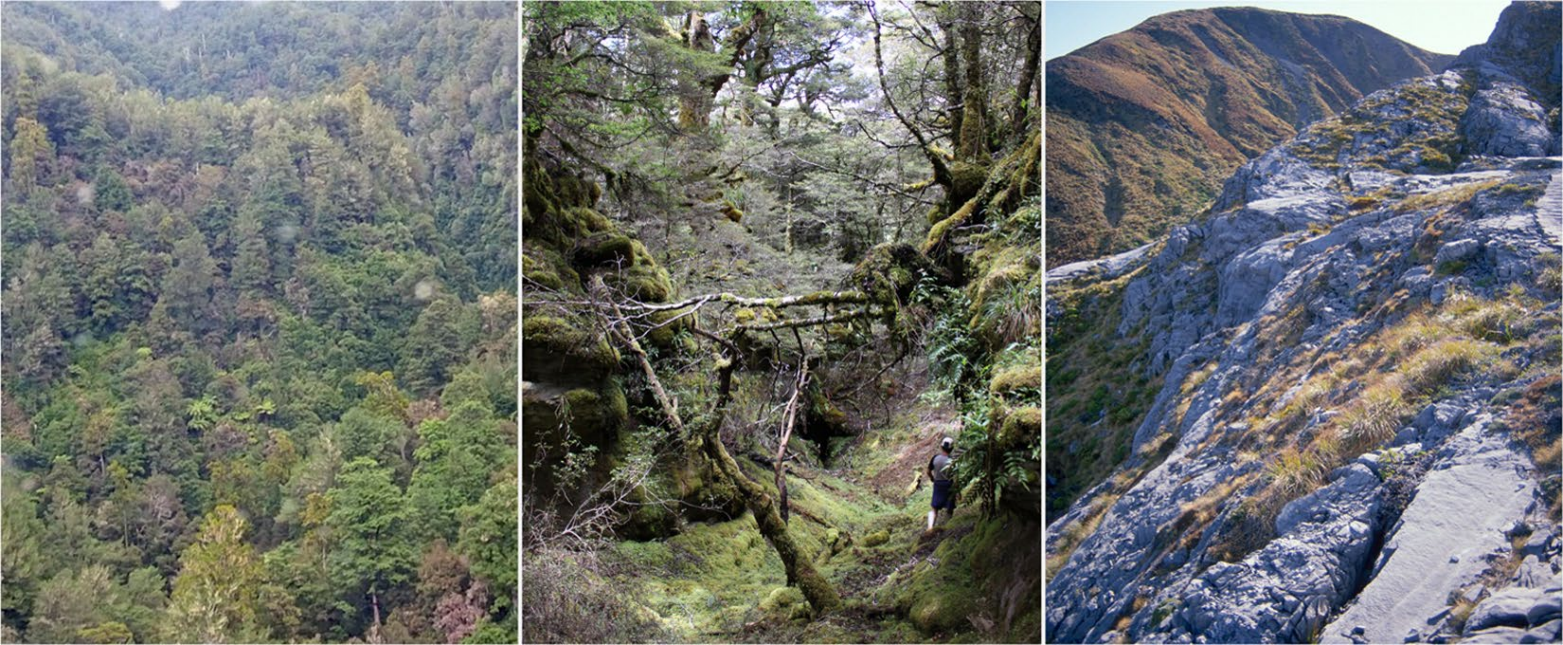
Representative vegetation types of Mount Arthur. Left: lowland broadleaf-podocarp forest near the lower entrance of Nettlebed cave at ca. 350 m asl (photo: J. Hellstrom). Centre: Open *Nothofagus* forest on limestone at ca. 950 m asl (photo: A. Hartland). Right: marble pavement and low alpine plants near the upper reaches of Nettlebed at ca. 1400 m asl (photo: J. Hellstrom).

An altitudinal transect from 300 to 1600 m asl was walked above Nettlebed Cave, with soil profiles and vegetation photographed at regular intervals (Table 2). Figure 2 shows three of the vegetation types encountered, contrasting the biologically productive low-altitude podocarp-broadleaf forest with alpine herbs and grasses above the treeline.

**Table 2:** Soil pits dug on the surface above or near to the cave passages below, arranged by altitude. O horizon thickness is of the upper humic horizon, and vegetation is as observed from the soil pit location.

Altitude (m)	O horizon (mm)	Vegetation
**South-eastern slopes**		
443	100	Broadleaf forest with dense understory and emergent podocarps
641	120	Mixed broadleaf - *Nothofagus* forest with dense understory
851	40	*Nothofagus* forest with open understory
1058	50	*Nothofagus* with sparse understory
1239	40	Stunted *Nothofagus* < 3 m high with sparse understory
1242	10	Alpine grassland with herbs and low shrubs
**Northern-western plateau**		
950	120	Tall *Nothofagus* forest with open understory

### U-Th geochronology

Samples of between 30 and 230 mg (median mass of 120 mg) were extracted from the sectioned cores using 0.5 to 0.8 mm tungsten carbide dental bits, by trenching around the desired sample under a film of flowing water after which a clean prism of calcite could be snapped off using tweezers. Typical stratigraphic (along-axis) extent of extracted samples was 2 to 4 mm, recorded for each sample to allow estimation of depth uncertainty.

Samples were dissolved in isolation using 1.5 M HNO_3_, spiked with a measured quantity of ^233^U-^229^Th or ^236^U-^233^U-^229^Th mixed synthetic isotopic tracer^21,22^ and allowed to equilibrate on a hotplate overnight. U and Th were separated from the carbonate matrix using Eichrom TRU ion exchange resin and established methods^23^, modified to collect U and Th in the same fraction using 0.1 M HCl–0.2 M HF^21^.

Mass spectrometric analysis was undertaken at The University of Melbourne using either a Nu Instruments Plasma MC-ICP-MS with standard collector block or a similar instrument with modified collector block placing its high-mass ion counter behind an electrostatic energy filter. ^229^Th/^230^Th and ^233^U/^234^U were measured simultaneously using the high- and low-mass SEM ion counters in each case^21^. Ion counter gain was determined using ion counter and Faraday measurements of ^235^U and ^229^Th for the standard collector block and ^236^U/^233^U and ^229^Th for the modified collector block^22^. Mass bias was determined using the bulk earth natural ^238^U/^235^U ratio of 137.88, this technique being relatively insensitive to error in this ratio due to the simultaneous measurement of ^229^Th/^230^Th and ^233^U/^234^U. Ion counter baselines were measured at half mass units and interpolated for ^234^U and ^230^Th^21^.

Initial ^230^Th/^232^Th activities were determined by stratigraphic constraint^24^ where possible, giving a value of 0.93 ± 0.08 for core MD3, the best-constrained speleothem. ^232^Th content in speleothems of the Ordovician Mount Arthur marble correlates with insoluble residue of white mica in the dating samples and is assumed to be relatively consistent throughout the marble caves (a larger uncertainty of ±0.2 is used where unconstrained). The flowstone samples from limestone host rock (Cores HC15-x) are allocated the same median value, but with ± 50% uncertainty. Calculated ages were corrected for initial ^230^Th/^238^U using measured ^232^Th/^238^U and stratigraphically-derived initial ^230^Th/^232^Th^24^ and the Cheng *et al*. 2013 decay constants^25^, with uncertainties calculated using 10,000-point Monte-Carlo numerical solutions of the age equation.

### Derivation of normalised growth probability records

Construction of time-resolved proxy records from speleothems requires some number of age determinations with their associated depths along the growth axis (with uncertainties for age and depth), from which a model of axial age can be derived as a function of depth, ideally also with an uncertainty envelope^26^. Any such model can be continuously differentiated to give most probable growth rate at any depth (and therefore at any time). As U-Th geochronology has become more affordable and more accessible, dating density has greatly increased, forcing a move away from linear interpolation between age determinations^14^ to more sophisticated Monte-Carlo-based procedures^27–29^.

Age-depth models (ADM) for Mount Arthur core samples were developed using the Finite Positive Growth Rate Model^30^, a Monte-Carlo technique which finds the most probable continuous series of age-depth line segments through a sequence of age determinations randomised according to their uncertainties. The model fit is subject to the constraints that growth rate should not change excessively in relative terms between adjacent age determinations (with respect to a user-controlled sensitivity parameter) and that it remains finite and positive for all segments of each iteration. Growth rate was then allowed to vary randomly between successive age determinations^27^ to account for “accordion effect” (error in interpolated age)^31^, for each iteration. 10,000 iterations of the model for each speleothem were sampled at ca. 0.5 mm depth intervals to determine median age at each depth and its uncertainty^27^.

To derive continuous estimates of relative probability of growth for a speleothem vs. time, the 10,000 Monte-Carlo age iterations from every 0.5 mm depth increment of its ADM were combined into a single histogram with 0.5 ka bin width. The form of this histogram is similar to a curve derived by inverting the median ADM to give depth vs. age and differentiating it to give median growth rate vs. time, but more accurately reflects growth probability near speleothem growth endpoints. The relative growth probability curves were then normalised such that maximum (usually interglacial) growth rate is equal to 1, unless the maximum growth rate was a transient spike in which case it was normalised to be 1.25 (a transient spike might reflect truly high growth rate, or it may be a short section of the ADM where growth rate is artificially high due to inversion of successive age determinations within their uncertainties). In two cases (the upper-most 2 mm of NB8 and lower-most 2 mm of HC15-1, both isolated by growth hiatuses) a single age determination for that interval was entered twice into the age model to better depict its age range in terms of growth probability.

## Results

**Figure 3:**
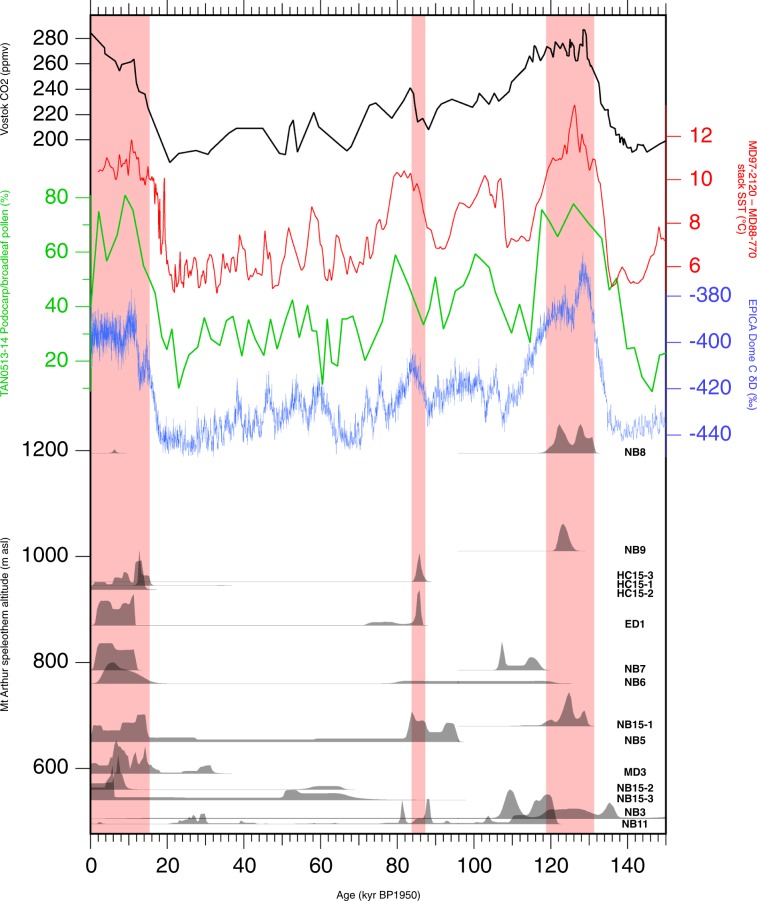
Mount Arthur flowstone core sample normalised relative growth probability vs. time, displayed with the baseline level of each curve arranged by the altitude of the surface above that flowstone. Sample names are at right. Red bars indicate growth intervals inferred to be similarly warm to or warmer than the present day. Shown for comparison are EPICA Dome C ice core 𝛿D^43^ on the AICC2012 Ice Age timescale^56^; TAN0513-14 marine core podocarp-hardwood tree pollen percentage^45^, west of South Island; MD97-2120 – MD88-770 SST stacked Southern Ocean SST record^44^; and the Vostok Ice core atmospheric CO_2_ record^57^ on the AICC2012 Gas Age timescale^56^.

284 U-Th age determinations were successfully undertaken, of which 234 had age corrections due to initial ^230^Th of their detrital component of less than 5%. Ages ranged between 0 and >550 ka (i.e. beyond the limit of U–Th dating), with 98% being between 0 ka and 250 ka (Supplementary Table 1). Only two core samples contain calcite that grew during the marine isotope stage (MIS) 7 interglacial, or older (not shown), whereas seven samples contained material from the last interglacial (MIS 5) and ten samples contained material from the Holocene (MIS 1). Figure 3 shows normalised growth probability curves for each speleothem, arranged according to altitude of the surface directly above. The two samples containing MIS 7-aged material, NB8 and NB9, both underlie surface altitudes of >1000 m asl, and also contain MIS 5-aged material. NB9 also grew prior to MIS 7, over at least two presumably interglacial periods prior to about 420 ka.

### Potential sources of growth interval bias

Increase in surface altitude over time due to tectonic uplift on the landscape would make past interglacial tree lines appear higher than they really were, and is difficult to constrain. The incision rate within the cave has been constrained to <0.40 m/ka by a magnetically-reversed speleothem ca. 300 m above the active phreatic level^16,17^ and can also be constrained to <0.31 m/ka by the 162 ka basal age of NB3, ca. 50 m above the active phreatic level. Incision rate within the cave system is ultimately constrained by that of the Pearse River gorge, downstream and east of Nettlebed Cave. Late Quaternary incision rates of New Zealand rivers have been shown to overstate late Quaternary tectonic uplift rates by a factor of ca. 1.5^32^, implying the tectonic uplift rate of Mount Arthur to be 0.2 m/ka or less which would correspond to ca. 25 m uplift since the MIS 5e interglacial and ca. 50 m since MIS 7e. Marble surfaces buried under soil at similar altitude to that above NB8 and NB9 have been found to have current day dissolution rates of only ca. 0.02 m/ka^33^, however these sites have been exposed to periglacial erosional processes well above the treeline for much of each of the past two glacial cycles. South Island montane erosion rates averaged over glacial and interglacial climates have been found to be broadly similar to local uplift rate over a wide range of uplift rates^34^, limiting surface altitude change above the Mount Arthur caves to at most a few tens of metres, in the context of the 710 metre altitudinal range under study.

As seen in the range of core sample basal ages (Table 1), the dataset is biased towards young material due to several processes, meaning it is difficult to assign relative significance to the number of flowstone samples which grew during each interglacial. The oldest flowstones are not present at the lowest cave levels as they cannot cover cave walls or floors until passages have been lifted above the active phreatic zone, affecting NB3, ED1, NB11, NB15-2 and NB15-3. Core samples NB5 and NB8 did not reach the base of their flowstones due to drill barrel limitations, and the deepest cores MD3 and NB15–1 contained intervals of dissolved calcite, presumably due to subsurface mixing corrosion where two or more carbonate-saturated solutions of different chemical composition become newly aggressive on mixing^35^. The HC-x samples formed close to the active stream level of a presumed to be relatively young limestone cave.

The CO_2_ content of poorly ventilated cave air can be much greater than that of the external atmosphere, leading to slower CO_2_ outgassing from drip water and reduced speleothem growth rate^9^. Caves may be ventilated by barometric pumping, external wind action and thermal chimney effects^36–38^, all of which apply to the caves of Mount Arthur where air movement can usually be observed through relatively constricted passage sections including into or out of rockfall blockages. Ventilation is likely to have further improved during glacial periods due to reduced vegetation and humus in and around surface openings and cannot be used to explain reduced speleothem growth rates at these times.

The Mount Arthur marble is heavily fractured and percolation water is found throughout the cave system, responding to changes in external precipitation on a seasonal basis^15^. As such it is assumed that waters feeding flowstone growth are sourced from close to directly above each site, with relatively little lateral travel.

### Growth intervals by altitude

The lowest-altitude speleothems were able to grow at almost any time during the last glacial cycle, whereas those underlying the highest altitude surfaces grew only during presumed warmer-than-present interglacial conditions. Core sample NB8 is one of the highest-surface-altitude flowstones known from the cave system (1210 m asl), while the many kilometres of known passage underlying higher slopes of Mount Arthur have minimal known flowstone development. At medium and high altitudes, variability of growth probability over time within individual speleothem cores also has an apparent relationship to global glacial-interglacial climate variability, with interglacial growth typically an order of magnitude greater than glacial growth rates, and abrupt increases in growth rate at glacial terminations (Fig. 3). The relationship of growth probability to external climate change is less clear at the lowest altitudes where there is presumably always forest vegetation and relatively well-developed soil above the cave. Other factors such as changes in precipitation or cave ventilation may have greater relative importance at low altitudes.

Soils were found to thin with increasing altitude, becoming more podzolised with thinner humic O horizons. Relatively fertile rendzic melanic (ER)^39^ soils were found at the two lowest altitude soil pits (Table 2; Fig. 1), grading into heavily leached orthic podzol soils (ZO)^39^ near and above the tree line. This corresponds to a vegetation change from tall broadleaf forest with emergent podocarp conifers and dense, diverse understory at lowest altitude, to low *Nothofagus* forest with open and less diverse understory at intermediate altitudes, to alpine grass and herb field above the tree line at ca. 1200–1300 m asl (Fig. 2). *Nothofagus* forest immediately below the tree line is stunted (ca. 3 metres) with little understory and podzolised soil.

### Timing of mid-high-altitude speleothem growth intervals

**250–215 ka**: highest-altitude flowstones NB8 and NB9 both grew during MIS 7, indicating warmer-than-present conditions at some time during that interglacial but neither flowstone gives a useable age range. The short MIS 7 section of NB8 is topped by an unconformity and the core sample did not reach its base. NB9 grew very slowly during MIS 7 with at least two hiatuses.

**131–119 ka**: highest-altitude flowstones NB8 and NB9 grew during MIS 5e as did mid-altitude flowstone NB15-1, indicating warmer-than-present conditions. Whilst NB9 grew slowly, NB8 grew 465 mm during this interval, and NB15-1 grew over 900 mm, more than twice the growth of any flowstone during the Holocene.

**119–107 ka**: medium-altitude flowstone NB7 grew during MIS 5d, but flowstones above 800 m asl did not, indicating cooler-than-present conditions. The two lowest-altitude flowstones NB3 and NB11 grew rapidly at times during this interval although should be interpreted with care as low-altitude flowstones are not clearly temperature-controlled.

**87–84 ka**: medium-altitude flowstones HC15-3 and ED-1 grew very rapidly during this brief interval corresponding to the peak of MIS 5a, but the higher-altitude flowstones did not grow, indicating conditions briefly similar to present.

**15.5–11.5 ka**: Late Glacial warming caused growth to start or rapidly accelerate in several flowstones at medium altitudes: HC15-1, HC15-2, NB5 and MD3. There is little difference in timing by altitude, implying the tree-line climbed very fast at ca. 15.5 ka to a near-present level.

**11.5–0 ka**: most of the flowstones studied began, resumed or accelerated in growth at or shortly after 11.5 ka, indicating the onset of Holocene conditions. The highest-altitude speleothem NB8 grew a very small amount, ca. 2 mm at ca. 7 ka, while NB9 remained inactive, indicating Holocene conditions were less favourable for forest expansion than either of the two preceding interglacial events. Four other flowstones, HC15-2, NB6, MD3 and NB15-2 grew relatively fast during the early– to mid–Holocene, suggesting an early Holocene optimum slightly warmer than present.

### Speleothem growth during glacial maxima

As many as three low-altitude Nettlebed Cave flowstones (NB5, NB15-2 and NB15-3) may have grown during the MIS 4 glacial maximum at 65.1 ± 5.4 (2σ)^40^, with NB15-3 appearing to have its highest growth probability during this interval, implying local tree line lowering of less than 500 m.

Only MD3 clearly grew through the entirety of the MIS 2 global LGM (24–18 ka)^41^ and the New Zealand MIS 2 extended LGM (eLGM; 29–19 ka)^42^ but two other low-altitude flowstones (NB11 and NB15-3) must have grown for at least part of both intervals, and one further flowstone (NB5) must have grown for at least part of the eLGM, together implying local tree line to have lowered by less than 600 m.

## Discussion

Figure 3 compares intervals of relatively high speleothem growth probability beneath different altitudes of Mount Arthur with other regional proxies for temperature over the last glacial cycle. The most striking feature of the flowstone growth record is that the two highest-altitude core samples grew during MIS 5e but not significantly during the Holocene. There is also an interval of growth at mid-altitude during MIS 5a and late MIS 2. The rank order of flowstone growth of these intervals (greatest during MIS 5e, less during MIS 1 and MIS 5a, and less still during late MIS 2) is broadly consistent with the EPICA Dome C ice core dD proxy^43^ for Antarctic temperature, where MIS 5e is the warmest event of the last glacial cycle, MIS 1 is the second warmest event, and late MIS 2 and MIS 5a are still cooler events. This suggests that flowstone growth at Mount Arthur over the last glacial cycle was strongly influenced by hemisphere-scale temperature change, through the effect of the latter on the upper altitude of forest growth.

Sea-surface temperature (SST) immediately west of South Island shows a similar pattern in which the warmest of the interglacials/interstadials is 5e, but MIS 5a and the Holocene are similarly warm^44^. This lends support to the impression gained here from the changing upper altitude of speleothem growth, that MIS 5a climates at ca. 83 ka were similarly warm and favourable for speleothem growth as those during the Holocene, in contrast to the EDC Antarctic dD record. The TAN0513-14 marine record of terrestrial pollen^45^ immediately west of South Island does not show MIS 5e as warmer than the Holocene, but subject to limitations of its resolution and tuned chronology is consistent with highest growing speleothem being an indicator of regional forest extent.

Speleothem growth has a clear relationship to soil and vegetation conditions above the cave, as demonstrated in other settings^10,11,46^. Flowstone growth of any age appears to have been restricted to cave passages beneath forest. By comparison, the extensive sections of the cave underlying montane shrub-, grass- and herb-lands with thin stony soils have negligible speleothem development and no significant flowstone growth. The speleothems able to grow during global glaciations currently underlie lowland podocarp/broadleaf forest with rich humic soils, and speleothems that grew or grow only during warm interstadial or interglacial episodes currently underlie *Nothofagus* beech forest, where soils become thinner, more leached and less humic with increasing altitude. The most sensitive flowstones are evidently those at intermediate to high altitudes, where the mountain surface above is swept by the treeline as it moves vertically in response to changing climates.

Flowstone growth at Mount Arthur indicates that soil and vegetation conditions at least as productive as those found somewhat below the current tree line persisted throughout the last glacial cycle, limiting tree line lowering to 500 m during the MIS 6 glacial maximum in New Zealand and 600 m during the MIS 2 LGM. This is consistent with reconstructed LGM vegetation to the east of Mount Arthur, of shrubland-grassland mosaic with patches of Nothofagus and rare conifers^47^. A range of possible past temperature lapse rates of between 4 and 7 °C per km of altitude have been suggested, for both islands of New Zealand^48,49^, giving local temperature reductions of 2–3.5 °C during MIS 4 and 2.4–4.2 °C during MIS 2.

The *Nothofagus* tree line in New Zealand corresponds to a coldest month temperature of ca. 0 °C^18^. Any change in seasonality of temperature during glacial climates would cause change in coldest month temperature to differ from change in MAT. A modelling study run using the United Kingdom Met Office HadAM3H and HadRM3H climate models found LGM temperature reductions of 3–4 °C for both winter (JJA) and summer (DJF) temperatures around Mount Arthur^50^ suggesting that treeline movement is proportional to MAT change in this setting. Reconstructed LGM SST change immediately east of South Island (SO136-GC3) was ca. 4 °C^44^, and reconstructed glacial equilibrium-line altitude lowering of ca. 965 m at Boulder Lake, 35 km north-northwest of Mount Arthur suggests cooling of 5–7 °C^51^. The speleothem-based treeline-lowering estimate of LGM temperature 2.4–4.2 °C cooler than the late Holocene is in the low- to mid-range of the climate modelling, marine SST and glacial equilibrium-line estimates.

Changes in mean annual precipitation affect speleothem drip rate, which can positively influence speleothem growth rate^1^, especially for speleothems in semi-arid settings^5^. At Mount Arthur speleothem growth is greatest at lowest altitudes, in inverse relationship to annual precipitation which is greatest on the upper slopes^19^, the opposite of expected for an annual precipitation control on growth rate. Climate and icefield modelling both suggest slightly drier conditions during the LGM^50,52^, consistent with Mg/Ca and ^234^U/^238^U records of Mount Arthur speleothem MD3^7^. Any growth rate effect of reduced precipitation would have its greatest impact at low altitudes where rainfall is currently at its lowest, rather than beneath the upper slopes where even a 25% reduction would leave annual precipitation of ca. 2000 mm^19^ and is not plausibly responsible for the absence of LGM speleothem growth there.

Atmospheric CO_2_ change should also be considered as an agent of tree line altitude change, expected to cause a reduction of water-use efficiency of plants during glaciations^53^, which would have the effect of minimising the temperature change required to explain the reconstructed tree line movement. However, this effect is at its weakest in cold environments with high water availability^53^ such as at Mount Arthur. Whilst the speleothem growth probability and atmospheric CO_2_ records are broadly in phase, detail such as an inverse relationship during the Holocene, and cessation of growth in the highest-altitude speleothems whilst CO_2_ remained near its maximum interglacial value, argues against an important role for CO_2_ in governing the altitude of speleothem growth at Mount Arthur over the last glacial cycle (Fig. 3).

It is more difficult to constrain tree line movement during warmer interglacial climates as it is not possible to know if either the highest currently growing speleothem or the highest speleothem growth corresponding to the previous warm interval has been located. NB9 and NB8 are the highest surface altitude flowstones that were located in Nettlebed or Exhaleair caves at the time of fieldwork; neither were active, although NB8 grew a very small amount during the mid-Holocene. The current upper surface altitude limit for flowstone growth seems likely to be in the 1000–1100 m range, which would require a minimum 100–200 m tree line altitude increase and at least 0.4–1.4 °C temperature increase for NB8 to grow during the Holocene optimum and earlier interglacials. Recently discovered passages linked to Nettlebed Cave include a section with flowstone beneath alpine terrain similar to that in Fig. 2 with a surface altitude of 1390 m asl, meaning it would have grown with a tree line at least 200–300 m higher than present under conditions at least 0.8–2.1 °C warmer. Reaching this site with drilling equipment will be a non-trivial task and the age of this growth event remains unknown for now.

The pronounced Late Glacial MIS 2 warming event inferred at 15.5 ka is in close agreement with the mean valley-floor deglaciation age of the nearby Cobb Valley (15 km to the northwest) at 15.6 ± 1.8 ka^54^ and begins at a similar phase of Antarctic warming^43^ to the MIS 5e flowstone growth event. The presumed initiation of full Holocene interglacial conditions, at ca. 11.5 ka, seen in the restarting of growth of medium-altitude NB7 and ED1, coincides with maximum Holocene Antarctic temperature^43^ and shortly follows the culmination of rapid post-glacial warming at Adelaide Tarn^55^, 35 km to the north-northwest.

## Supplementary Information

Supplementary Information.

## Data Availability

All data generated or analysed during this study are included in this published article (and its Supplementary Information files).
