# Supplementary material for: Speleothem growth intervals reflect New Zealand montane vegetation response to temperature change over the last glacial cycle
Source: Sci Rep. 2020 Feb 12;10:2492. doi: 10.1038/s41598-020-58317-8 (PMC7015920; doi:10.1038/s41598-020-58317-8)
Supplement: Supplementary file 1 — Supplementary Information. [file 41598_2020_58317_MOESM1_ESM.pdf]

Supplementary data for "Speleothem growth intervals reflect New Zealand montane vegetation response to temperature change over the last glacial cycle"

John Hellstrom*[1], Kale Sniderman[1], Russell Drysdale[2,3], Isabelle Couchoud[3,2], Adam Hartland[4], Andrew Pearson[4], Petra Bajo[5]

1. School of Earth Sciences, University of Melbourne, Victoria, 3010, Australia
2. School of Geography, University of Melbourne, Victoria, 3010, Australia
3. Laboratoire EDYTEM, CNRS, Université Savoie Mont Blanc, Université Grenoble Alpes, Chambéry, France
4. Environmental Research Institute, School of Science, University of Waikato, Private Bag 3105, Hamilton, 3240, New Zealand
5. Croatian Geological Survey, Sachsova 2, 10 000 Zagreb, Croatia

*Corresponding author. j.hellstrom@unimelb.edu.au

A

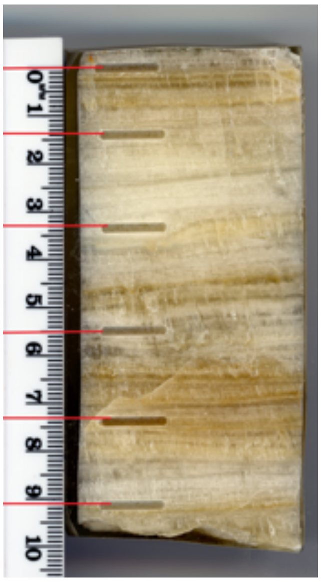

B

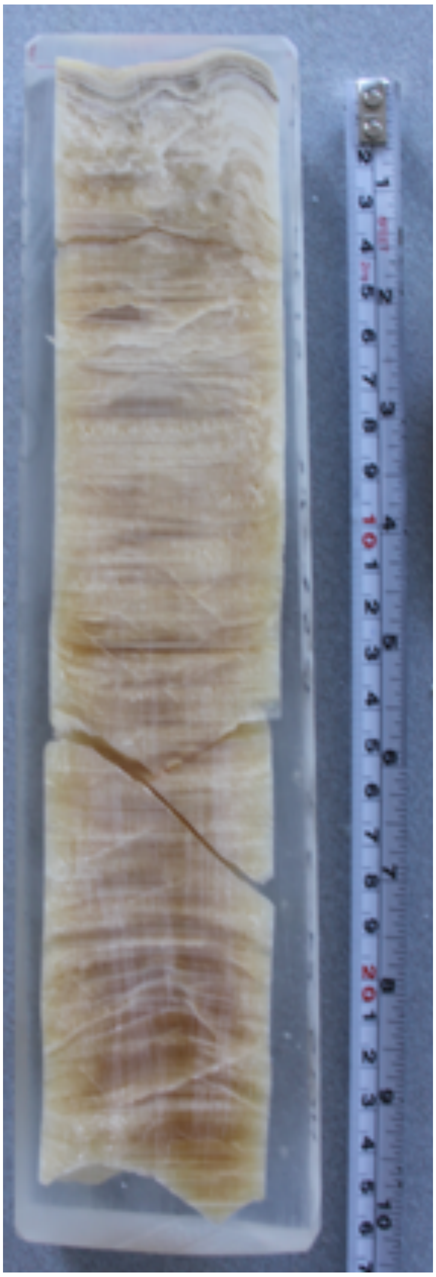

C

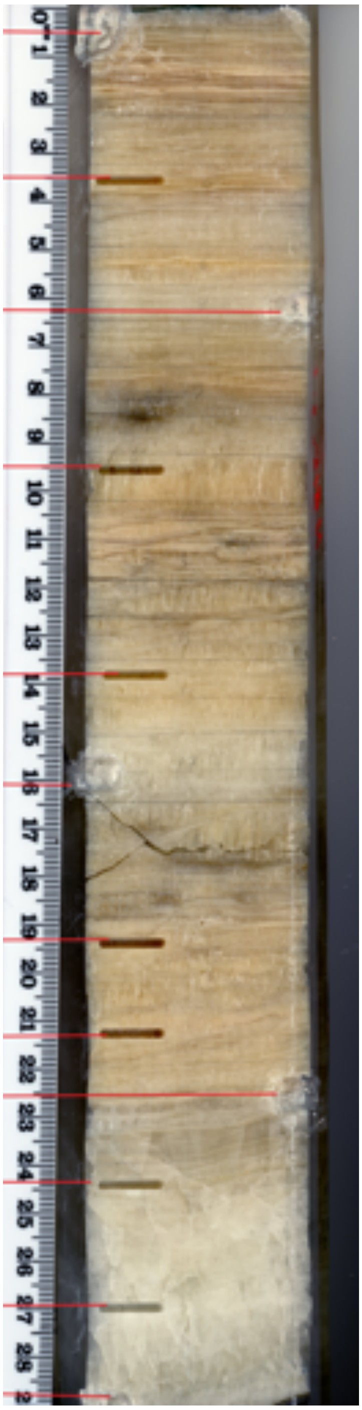

D

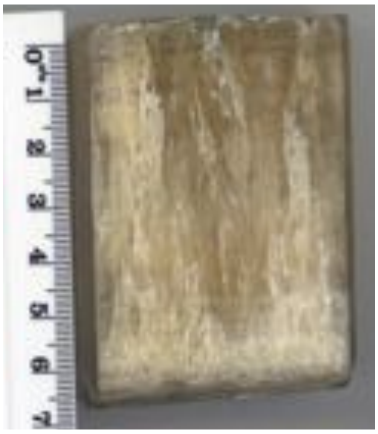

Supplementary figure 1. The upper sections of cores (A) NB15-2, (B) HC15-3, (C)NB15-3 and (D) NB15-1, illustrating representative variation in fabric and organic and detrital content between flowstone beneath Mt Arthur.

Photos: A. Pearson

Supplementary table 1. Mt Arthur flowstone U-Th analyses

Sample	Depth(mm)	U(ngg^{-1})	[^{230}Th/^{238}U]^{a}	[^{234}U/^{238}U]^{a}	[^{232}Th/^{238}U]	[^{230}Th/^{232}Th]	Age(ka BP)^{b}	[^{234}U/^{238}U]$_{i}^{c}$
**MD3**								
MD3.10 top	1.5(1.5)	52	0.0296(15)	1.3245(38)	0.0289101(2073)	1.0	0.163(0.230)	1.3247(39)
MD3.15	38.0(2.5)	68	0.0252(07)	1.3339(26)	0.0009122(224)	28	1.944(0.058)	1.3358(26)
MD3.16	89.5(2.0)	n.a.	0.0618(07)	1.3235(28)	0.0002620(18)	236	5.120(0.062)	1.3282(29)
MD3.14	130.0(2.0)	n.a.	0.0784(11)	1.3407(27)	0.0016666(71)	47	6.365(0.095)	1.3469(27)
MD3.9	181.5(0.5)	60	0.0864(26)	1.3398(117)	0.001184(65)	73	7.10(0.23)	1.3467(120)
MD3.17	244.0(3.5)	n.a.	0.0992(15)	1.3414(31)	0.000445(04)	223	8.25(0.13)	1.3495(31)
*MD3.8	293.0(1.5)	53.9	0.1183(18)	1.3469(33)	0.013318(236)	8.9	8.92(0.18)	1.3558(34)
*MD3b-1/upper	293.0(1.0)	47.9	0.1233(23)	1.3449(41)	0.007280(26)	17	9.83(0.21)	1.3547(42)
MD3b-2	295.8(0.8)	n.a.	0.1172(18)	1.3501(37)	0.005484(184)	21	9.39(0.16)	1.3595(38)
MD3b-3	298.3(0.8)	41	0.1153(24)	1.3523(42)	0.001513(07)	76	9.50(0.22)	1.3620(43)
MD3b-4	300.8(0.8)	n.a.	0.1143(18)	1.3585(41)	0.00234(10)	49	9.31(0.16)	1.3681(42)
MD3b-7	308.8(0.8)	59	0.1187(18)	1.3522(43)	0.00141(05)	84	9.8(0.2)	1.3621(43)
MD3b-12	312.3(0.8)	n.a.	0.1207(18)	1.3506(44)	0.0021(01)	58	9.9(0.2)	1.3606(45)
*MD3.18	317.0(2.0)	n.a.	0.1213(15)	1.3594(27)	0.00021(00)	581	10.07(0.13)	1.3698(27)
MD3b-11	318.8(0.8)	51.1	0.1213(15)	1.3606(34)	0.001355(53)	90	9.98(0.13)	1.3710(35)
MD3b-13	323.8(0.8)	n.a.	0.1235(25)	1.3612(54)	0.001826(53)	68	10.13(0.22)	1.3718(55)
MD3b-15	328.8(0.8)	n.a.	0.1299(15)	1.3554(34)	0.00093(00)	139	10.81(0.14)	1.3665(35)
MD3b-16	331.3(0.8)	83.1	0.1307(17)	1.3564(31)	0.000426(02)	307	10.91(0.15)	1.3676(32)
MD3b-18	336.3(0.8)	n.a.	0.1329(14)	1.3549(31)	0.00115(04)	116	11.06(0.13)	1.3662(32)
MD3b-20	341.3(0.8)	88	0.1350(14)	1.3397(30)	0.00058(01)	234.2	11.42(0.13)	1.3509(31)
*MD3.11	345.5(2.0)	82	0.1429(12)	1.3383(22)	0.00051(00)	280	12.15(0.11)	1.3502(23)
MD3b-24	351.3(0.8)	n.a.	0.1363(13)	1.3483(30)	0.00054(02)	254.5	11.46(0.12)	1.360(03)
MD3b-26	356.3(0.8)	115	0.1418(21)	1.3645(38)	0.00106(00)	133.6	11.76(0.19)	1.377(04)
MD3b-28	361.3(0.8)	n.a.	0.1417(11)	1.3641(26)	0.00045(02)	317	11.81(0.10)	1.3765(27)
MD3b-29	363.8(0.8)	n.a.	0.1424(10)	1.3627(27)	0.00037(01)	381.5	11.89(0.09)	1.3751(28)
MD3b-32	371.3(0.8)	97	0.1450(10)	1.3652(28)	0.000268(06)	542	12.10(0.09)	1.3779(29)
MD3b-34	376.3(0.3)	n.a.	0.1457(15)	1.3655(36)	0.0003(00)	438.9	12.16(0.14)	1.3783(37)
MD3b-36	380.8(0.8)	n.a.	0.1466(15)	1.3579(37)	0.000594(03)	247	12.29(0.14)	1.3706(38)
MD3b-37	383.3(0.8)	n.a.	0.1505(12)	1.3611(32)	0.00203(05)	74.0	12.50(0.11)	1.3741(33)
MD3b-39	388.3(0.8)	n.a.	0.1523(10)	1.3638(32)	0.00082(02)	187	12.72(0.10)	1.3772(32)
MD3b-41	393.3(0.8)	76	0.1548(11)	1.3605(26)	0.00037(01)	424	13.01(0.10)	1.3741(27)
MD3b-42	395.8(0.8)	n.a.	0.1568(19)	1.3564(29)	0.000214(10)	732	13.25(0.18)	1.3700(30)
MD3b-44	400.8(0.8)	n.a.	0.1564(13)	1.3536(26)	0.000517(16)	302	13.22(0.12)	1.3671(27)
MD3b-46	405.8(0.8)	n.a.	0.1606(09)	1.3656(26)	0.00155(04)	104	13.39(0.09)	1.3797(27)
MD3b-47	408.3(0.8)	76	0.1644(13)	1.3740(25)	0.00109(02)	151	13.67(0.12)	1.3888(26)
MD3b-50	415.8(0.8)	n.a.	0.1669(11)	1.3861(27)	0.00073(02)	228	13.79(0.10)	1.4015(28)
*MD3.7	418.0(2.0)	72	0.1650(13)	1.3721(30)	0.00686(12)	24	13.33(0.13)	1.3864(31)
MD3b-53	423.3(0.8)	105	0.1715(12)	1.3865(25)	0.00080(03)	214	14.19(0.11)	1.4024(26)
MD3b-56	430.8(0.8)	n.a.	0.1720(13)	1.3892(26)	0.00054(02)	317	14.22(0.12)	1.4052(27)
MD3b-59	438.3(0.8)	n.a.	0.1765(13)	1.3932(32)	0.00053(02)	331	14.58(0.12)	1.4098(33)
MD3b-60	440.8(0.8)	107	0.1804(14)	1.3969(33)	0.00554(14)	33	14.52(0.14)	1.4136(34)
MD3b-63	453.3(0.8)	93	0.1835(20)	1.3948(39)	0.00776(14)	24	14.66(0.19)	1.4116(41)
MD3b-64	455.8(0.8)	n.a.	0.1800(21)	1.3953(31)	0.00092(02)	195	14.83(0.19)	1.4123(32)
*MD3.6	457.0(1.5)	131	0.1930(16)	1.3929(30)	0.02020(79)	10	14.63(0.20)	1.4095(31)
MD3b-65	458.3(0.8)	n.a.	0.1832(17)	1.3932(30)	0.00544(19)	34	14.82(0.16)	1.4101(31)
*MD3.5	461.5(1.5)	90	0.1782(21)	1.3920(40)	0.00084(01)	213	14.72(0.19)	1.4086(41)
MD3b-67	463.3(0.8)	76	0.1845(19)	1.3986(31)	0.00076(02)	243	15.21(0.17)	1.4161(32)
MD3b-69	468.3(0.8)	n.a.	0.1870(20)	1.3999(42)	0.00191(06)	98	15.33(0.18)	1.4177(43)
MD3b-70	470.8(0.8)	n.a.	0.1888(25)	1.3995(33)	0.00470(12)	40	15.30(0.23)	1.4172(34)
MD3b-71	473.3(0.8)	73	0.1972(11)	1.3977(29)	0.00659(16)	30	15.93(0.11)	1.4161(30)
*MD3.12R	478.0(2.5)	n.a.	0.1982(21)	1.3983(40)	0.00159(02)	125	16.37(0.20)	1.4172(42)
MD3b-73	478.3(0.8)	93	0.1933(14)	1.3962(30)	0.00219(07)	88	15.92(0.13)	1.4145(31)
MD3.4	490.5(2.5)	80	0.2039(11)	1.3962(23)	0.00103(01)	198	16.95(0.10)	1.4157(24)
MD3.19	500.0(2.0)	162	0.2196(09)	1.3973(28)	0.00360(05)	61	18.17(0.09)	1.4183(29)
MD3.20	512.0(2.5)	n.a.	0.2797(16)	1.3740(28)	0.00467(02)	60	24.16(0.17)	1.4005(29)
*MD3.3	531.5(1.0)	160	0.3109(21)	1.3810(31)	0.01112(22)	28	26.62(0.23)	1.4108(33)
MD3.21	533.0(2.0)	125	0.3221(16)	1.3786(25)	0.00252(04)	128	28.41(0.17)	1.4103(27)
MD3.2	553.5(2.0)	93	0.3356(10)	1.3775(25)	0.00109(01)	308	29.91(0.12)	1.4108(27)
MD3.1	580.0(1.5)	77	0.3506(18)	1.3720(28)	0.00241(03)	145	31.50(0.20)	1.4067(29)
MD3 base-1	581.5(2.0)	104	0.3669(27)	1.3796(28)	0.02028(06)	18	31.70(0.32)	1.4152(30)
**ED1**								
ED1-13	1.5(1.0)	80	0.0201(10)	1.2358(35)	0.00332(16)	6.1	1.45(0.11)	1.2368(35)
ED1-15	10.3(1.8)	n.a.	0.0252(11)	1.2416(29)	0.00063(00)	40	2.12(0.10)	1.2431(29)
ED1.12	72.0(2.0)	98	0.0635(09)	1.2460(27)	0.00397(11)	16	5.31(0.11)	1.2497(27)
ED1-11	104.5(1.5)	89	0.0967(10)	1.2471(26)	0.01024(17)	9	7.88(0.20)	1.2527(26)
ED1-10	135.5(2.0)	95	0.1072(28)	1.2435(29)	0.00095(02)	113	9.66(0.27)	1.2503(29)
ED1-9	156.5(1.5)	73	0.1208(21)	1.2361(36)	0.00233(04)	52	10.92(0.21)	1.2435(36)
ED1-8	170.0(2.0)	79	0.1353(19)	1.2112(26)	0.01969(28)	7	11.16(0.40)	1.2180(27)
ED1.14	171.5(1.5)	n.a.	0.1240(19)	1.2467(31)	0.00108(01)	115	11.24(0.19)	1.2547(32)
ED1-7	174.0(2.0)	22	0.5991(47)	1.1964(32)	0.02305(20)	26	72.05(0.95)	1.2407(38)
ED1-6	183.5(1.5)	31	0.5861(34)	1.1365(26)	0.01688(16)	35	76.11(0.77)	1.1692(31)
ED1-5	187.0(2.0)	20	0.6121(83)	1.1804(82)	0.00381(22)	161	77.51(1.72)	1.2245(98)
ED1-4	193.0(2.0)	49	0.6486(55)	1.1768(45)	0.06309(73)	10	79.73(1.68)	1.2214(54)
ED1-3	198.0(2.0)	270	0.6375(31)	1.1202(22)	0.05382(65)	12	85.10(1.30)	1.1529(27)
ED1-2	218.0(2.0)	24	0.6768(57)	1.2143(40)	0.00214(11)	316	85.94(1.17)	1.2732(48)
ED1-1 BASE	232.0(1.5)	35	0.6658(41)	1.1980(35)	0.00711(10)	94	85.34(0.88)	1.2520(42)
**NB3**								
NB3T	1.5(1.5)	n.a.	0.3065(81)	1.1764(47)	0.21230(1114)	1	12.78(4.95)	1.1829(54)
NB3-5	47.5(2.0)	n.a.	0.7132(31)	1.1126(33)	0.00954(08)	75	108.10(1.02)	1.1527(42)
NB3-2.22	136.0(3.0)	102	0.7405(33)	1.1007(20)	0.07586(86)	10	111.74(1.86)	1.1381(27)
NB3.A	143.0(2.0)	54	0.7438(45)	1.0960(24)	0.02559(16)	29	118.37(1.49)	1.1341(32)
NB3-2.21	159.0(1.5)	48	0.7560(49)	1.0942(31)	0.04377(51)	17	120.72(1.85)	1.1325(41)
NB3-2.20	163.0(2.0)	64	0.7790(31)	1.0895(22)	0.11620(136)	7	122.15(2.74)	1.1264(31)
NB3-2.19	183.0(2.0)	66	0.8527(67)	1.0845(24)	0.34576(354)	2	125.44(9.93)	1.1204(46)
NB3.B	231.0(1.5)	59	0.8103(108)	1.1039(42)	0.11061(173)	7	129.36(4.50)	1.1497(58)
NB3-2.16	235.0(1.5)	82	0.8144(44)	1.0990(27)	0.13637(124)	6	129.58(3.41)	1.1427(38)
NB3-2.15	238.0(2.0)	71	0.8145(29)	1.1084(24)	0.05136(90)	16	135.10(1.58)	1.1587(33)
NB3-2.15	238.0(2.0)	62	0.8073(36)	1.1096(26)	0.07142(33)	11	130.51(1.97)	1.1585(35)
NB3-2.14	244.0(2.0)	44	0.8141(38)	1.1287(33)	0.00889(10)	92	133.25(1.45)	1.1875(44)
NB3-6	246.5(2.0)	n.a.	0.8052(95)	1.1219(41)	0.00389(05)	207	132.67(3.18)	1.1773(56)
NB3-2.13	247.0(2.0)	63	0.8296(43)	1.1219(26)	0.05441(60)	15	136.14(1.95)	1.1790(36)
NB3-2.12b	250.0(1.5)	79	0.8217(42)	1.1182(37)	0.10748(114)	8	129.57(2.81)	1.1704(50)
NB3-2.12a	254.0(1.5)	90	0.8445(59)	1.1189(21)	0.21678(173)	4	126.38(5.31)	1.1699(37)
NB8-23c	255.5(1.8)	45	1.0011(48)	1.4056(37)	0.00337(04)	297	124.28(1.22)	1.5761(46)
NB3-2.12	259.0(2.0)	65	0.8294(39)	1.1194(21)	0.05469(106)	15	136.75(1.82)	1.1757(29)
NB3-2.11	264.0(2.0)	57	0.8150(34)	1.1199(23)	0.03136(35)	26	133.89(1.42)	1.1750(31)

NB3-2.10	269.0(2.0)	70	0.8217(33)	1.1185(21)	0.03478(38)	24	136.18(1.41)	1.1741(29)
NB3-2.09	273.0(2.5)	74	0.8163(29)	1.1181(20)	0.00938(11)	87	136.74(1.11)	1.1738(27)
NB3-2.08	278.0(2.0)	69	0.8181(31)	1.1166(22)	0.01626(21)	50	137.15(1.23)	1.1717(30)
NB3-2.08a	278.5(1.5)	67	0.8131(32)	1.1190(25)	0.01837(13)	44	134.67(1.28)	1.1740(33)
NB3.4	280.0(2.0)	n.a.	0.8039(32)	1.1112(23)	0.00502(12)	160	134.95(1.20)	1.1628(31)
NB3-2.07	281.5(2.0)	76	0.8522(22)	1.1105(28)	0.16417(497)	5	137.17(3.83)	1.1628(41)
*NB3.C	288.0(2.0)	47	0.8542(75)	1.1221(38)	0.04119(39)	21	145.84(3.06)	1.1843(53)
NB3-C-a	288.0(2.0)	54	0.8394(63)	1.1257(50)	0.02958(20)	28	140.57(2.68)	1.1869(68)
NB3-2.03	293.5(2.0)	27	0.8585(54)	1.1143(34)	0.02252(38)	38	151.66(2.40)	1.1754(48)
*NB3-3	295.8(1.8)	n.a.	0.8512(63)	1.1170(40)	0.01825(17)	47	148.40(2.70)	1.1779(55)
NB3-2.04	298.0(2.0)	21	0.8592(61)	1.1127(29)	0.00831(17)	103	153.70(2.55)	1.1739(41)
NB3-2.06	300.8(2.5)	27	0.8595(34)	1.1026(26)	0.04199(72)	20	154.39(1.87)	1.1587(37)
NB3-2.05	308.8(2.5)	32	0.8585(53)	1.1029(32)	0.03797(50)	23	154.22(2.51)	1.1591(45)
NB3-2.02	322.0(2.5)	30	0.8566(64)	1.1075(34)	0.05050(173)	17	150.72(2.93)	1.1645(49)
NB3-2.01	351.0(2.5)	24	0.8673(44)	1.1096(33)	0.00875(17)	99	157.92(2.13)	1.1712(46)
NB3.1B	356.8(1.8)	33	0.8929(78)	1.0994(40)	0.12531(98)	7	162.25(4.81)	1.1571(58)
**NB5**								
NB5T	1.3(1.0)	n.a.	0.0040(07)	0.9791(22)	0.00054(02)	7	0.33(0.08)	0.9791(22)
NB5-10	33.0(2.0)	n.a.	0.0291(04)	1.0598(24)	0.00089(02)	33	2.88(0.05)	1.0603(24)
NB5-4	76.5(2.5)	n.a.	0.0748(04)	1.0477(18)	0.00029(01)	261	7.98(0.05)	1.0488(19)
NB5-5	133.5(3.0)	n.a.	0.1169(08)	1.1170(20)	0.00032(01)	361	11.94(0.09)	1.1210(21)
NB5-6	187.0(2.0)	n.a.	0.1460(06)	1.1462(22)	0.00210(07)	70	14.57(0.08)	1.1523(23)
NB5-1	228.0(3.0)	581	0.2370(09)	1.0589(18)	0.00118(03)	201	27.38(0.13)	1.0637(19)
NB5-7	280.0(5.0)	n.a.	0.4484(13)	1.0653(19)	0.00668(12)	67	58.49(0.30)	1.0770(22)
NB5-8	339.0(1.5)	n.a.	0.5885(26)	1.0713(22)	0.02487(105)	24	83.51(0.82)	1.0903(27)
NB5-3	364.0(1.5)	n.a.	0.5898(36)	1.0660(38)	0.03488(152)	17	83.54(1.20)	1.0836(47)
NB5-2	419.5(1.5)	387	0.5803(21)	1.0425(21)	0.00443(10)	131	87.56(0.58)	1.0544(26)
NB5-9	451.5(1.5)	n.a.	0.6040(19)	1.0507(18)	0.00356(07)	170	92.01(0.54)	1.0657(23)
NB5 BASE	495.0(2.5)	387	0.6148(18)	1.0445(21)	0.00852(11)	72	95.13(0.59)	1.0582(27)
**NB6**								
NB6T	1.0(1.0)	n.a.	0.1140(25)	1.1113(36)	0.08957(136)	1	3.24(1.94)	1.1123(37)
NB6.3	47.5(2.0)	n.a.	0.1035(22)	1.1246(23)	0.03643(44)	3	7.12(0.76)	1.1271(23)
NB6.1	102.5(2.0)	38	0.3015(28)	1.1446(29)	0.19634(161)	2	14.26(4.42)	1.1506(36)
NB6.4	133.0(2.3)	n.a.	0.6015(28)	1.0916(23)	0.05222(48)	12	81.14(1.28)	1.1152(28)
NB6.2	196.0(2.5)	58	0.7346(37)	1.0718(22)	0.04217(42)	17	119.64(1.55)	1.1007(30)
**NB7**								
NB7T	1.3(1.0)	n.a.	0.0180(40)	1.1326(25)	0.00496(04)	4	1.24(0.40)	1.1331(25)
NB7-3	82.5(1.5)	n.a.	0.0640(08)	1.1069(21)	0.00356(03)	18	6.10(0.11)	1.1088(22)
NB7-1	152.0(1.5)	114	0.1166(12)	1.1331(26)	0.00059(01)	196	11.70(0.13)	1.1376(27)
NB7-4	174.0(2.0)	n.a.	0.6894(91)	1.0824(23)	0.00226(08)	305	108.21(2.46)	1.1118(31)
NB7-2	192.5(1.5)	121	0.6818(27)	1.0753(23)	0.00369(04)	185	107.39(0.84)	1.1020(30)
NB7-5	213.0(1.5)	n.a.	0.7022(19)	1.0685(21)	0.00874(07)	80	113.91(0.73)	1.0945(28)
NB7 BASE	239.0(3.0)	100	0.7230(39)	1.0681(25)	0.04153(114)	17	117.01(1.57)	1.0948(33)
**NB8**								
NB8.19	0.8(0.8)	44	0.1252(59)	1.5586(101)	0.04316(28)	3	6.19(0.76)	1.5685(102)
NB8-8	8.5(2.0)	47	0.9599(58)	1.3820(30)	0.00792(12)	121	119.08(1.35)	1.5347(39)
NB8-9	34.5(2.0)	37	0.9663(75)	1.3764(34)	0.00563(10)	172	121.54(1.72)	1.5306(47)
NB8-10	56.0(2.5)	50	0.9725(75)	1.3758(43)	0.00196(04)	496	123.24(1.81)	1.5322(55)
NB8-6	59.5(1.5)	81	0.9675(57)	1.3738(31)	0.00253(04)	382	122.44(1.37)	1.5282(41)
NB8-7	131.0(3.0)	94	0.9771(39)	1.3863(37)	0.00082(01)	1185	122.52(1.05)	1.5460(45)
NB8.4	220.0(2.0)	30	0.9908(93)	1.3949(77)	0.00326(09)	304	123.88(2.39)	1.5602(94)
NB8.11	252.0(2.0)	46	1.0130(61)	1.4032(36)	0.00245(02)	413	127.38(1.51)	1.5777(47)
NB8.12	284.0(2.0)	80	1.0098(38)	1.4016(33)	0.00086(02)	1177	127.05(1.03)	1.5749(40)
NB8.13	324.5(1.5)	55	1.0286(82)	1.4122(31)	0.00501(14)	205	129.07(1.92)	1.5934(47)
NB8.14	363.0(2.0)	53	1.0203(78)	1.4170(41)	0.00116(03)	882	126.64(1.84)	1.5962(54)
NB8-3	401.0(3.0)	35	1.0406(54)	1.4238(32)	0.00443(14)	235	129.70(1.34)	1.6112(42)
NB8.15	436.5(3.0)	40	1.0271(115)	1.4241(31)	0.00443(13)	232	126.68(2.54)	1.6065(56)
NB8.18	450.0(3.0)	32	1.0515(56)	1.4243(46)	0.00112(01)	941	132.27(1.55)	1.6164(56)
NB8-JH2.2	463.0(2.0)	28	1.0532(56)	1.4288(36)	0.00947(09)	111	131.29(1.43)	1.6213(46)
NB8-JH2.1	465.0(2.0)	35	1.0539(43)	1.4344(36)	0.01471(18)	72	130.08(1.19)	1.6272(44)
NB8.17	489.0(2.0)	45	1.2100(49)	1.2709(43)	0.00311(02)	389	249.58(4.89)	1.5480(63)
NB8.20	492.5(1.5)	n.a.	1.2081(51)	1.2752(27)	0.00231(01)	523	244.95(4.03)	1.5495(58)
**NB9**								
NB9.6 TOP	1.0(1.0)	37	0.8107(44)	1.1730(33)	0.00364(16)	223	122.35(1.38)	1.2444(42)
NB9.9	15.5(1.5)	15	0.8263(86)	1.1886(67)	0.00595(13)	139	123.08(2.70)	1.2669(87)
NB9.10	33.0(1.5)	32	0.8420(79)	1.2011(33)	0.00483(12)	174	124.74(2.24)	1.2860(45)
NB9.20R	35.5(0.8)	n.a.	0.9654(50)	1.0973(39)	0.00423(03)	228	214.57(4.31)	1.1783(60)
NB9.20	35.5(0.8)	79	0.9652(30)	1.0952(27)	0.00183(03)	526	216.16(2.84)	1.1752(41)
NB9.11	39.0(1.5)	51	1.0272(64)	1.1317(31)	0.00538(11)	191	231.30(5.28)	1.2530(53)
NB9.12	60.0(2.0)	19	1.0174(62)	1.1230(29)	0.00168(05)	605	231.55(5.22)	1.2365(50)
NB9.13	72.0(1.5)	69	1.0241(67)	1.1191(25)	0.00752(12)	136	239.58(5.83)	1.2342(49)
NB9-4	75.0(2.0)	67	1.0254(46)	1.1186(35)	0.00144(01)	713	241.56(4.94)	1.2345(53)
NB9.5	85.5(2.0)	107	1.0556(55)	1.0509(33)	0.00179(03)	590	467.04(49.18)	1.1904(214)
NB9.21R	88.5(3.0)	106	1.0560(31)	1.0524(25)	0.00119(01)	888	455.49(27.85)	1.1895(103)
NB9.14	96.0(2.0)	192	1.0646(32)	1.0512(21)	0.00161(03)	659	545.08(60.92)	1.2391(371)
NB9.15	101.0(1.5)	86	1.0656(47)	1.0500(23)	0.01548(16)	69	594.79(140.47)	1.2737(1485)
NB9.16	116.5(2.0)	147	1.0662(28)	1.0487(22)	0.01007(12)	106	651.78(159.40)	1.3154(2041)
NB9.17	143.0(1.5)	57	1.0396(41)	1.0298(18)	0.00717(10)	145	658.43(196.02)	1.2003(1724)
**NB11**								
NB11-13.1	1.0(0.9)	n.a.	0.0263(12)	1.0940(28)	0.00250(02)	11	2.36(0.13)	1.0946(28)
NB11-13.3	1.0(1.5)	n.a.	0.0240(08)	1.0922(31)	0.00238(02)	10	2.14(0.09)	1.0928(31)
NB11-13.4	1.0(1.5)	n.a.	0.0356(10)	1.0912(30)	0.00418(04)	9	3.16(0.13)	1.0920(30)
NB11-14.1	4.0(0.9)	n.a.	0.1943(18)	1.0966(28)	0.00115(01)	169	21.04(0.23)	1.1025(29)
NB11-1a	7.0(0.9)	60	0.2121(19)	1.0988(28)	0.00090(01)	236	23.16(0.24)	1.1055(30)
NB11-14.3	7.0(1.5)	n.a.	0.2107(14)	1.0937(23)	0.00122(01)	173	23.09(0.18)	1.1000(25)
NB11-14.4	7.0(1.5)	n.a.	0.2091(12)	1.0933(27)	0.00057(01)	365	22.96(0.16)	1.0996(29)
NB11-14.2	11.0(1.0)	n.a.	0.2184(19)	1.0913(19)	0.00021(00)	1018	24.19(0.24)	1.0978(20)
NB11-15.1	17.5(1.4)	n.a.	0.2292(21)	1.0930(24)	0.00028(00)	833	25.49(0.27)	1.0999(26)
NB11-15.2	18.5(1.4)	n.a.	0.2296(15)	1.0917(18)	0.00013(00)	1765	25.58(0.20)	1.0986(19)
NB11-16.1	24.0(0.9)	n.a.	0.2345(19)	1.0915(35)	0.00042(00)	563	26.18(0.26)	1.0985(37)
NB11-16.2	24.5(1.5)	n.a.	0.2391(17)	1.0974(18)	0.00042(00)	567	26.59(0.22)	1.1050(19)
NB11-17.1	32.0(2.0)	n.a.	0.2431(15)	1.0974(24)	0.00041(01)	600	27.10(0.20)	1.1052(25)
NB11-17.2	33.5(1.4)	n.a.	0.2434(11)	1.0943(20)	0.00033(00)	748	27.24(0.15)	1.1019(22)
NB11-18.2	37.1(0.9)	n.a.	0.2521(18)	1.0909(25)	0.00032(01)	780	28.45(0.25)	1.0985(27)
NB11-7	40.0(2.0)	101	0.2548(19)	1.0970(26)	0.00025(00)	1036	28.62(0.26)	1.1052(28)

NB11-19.2	44.0(2.0)	n.a.	0.2569(22)	1.0932(18)	0.00029(00)	892	29.01(0.29)	1.1012(19)
NB11-8	50.5(1.5)	96	0.2606(18)	1.0905(27)	0.00013(00)	1961	29.58(0.25)	1.0984(29)
NB11-20.2	51.5(1.5)	n.a.	0.2596(16)	1.0879(23)	0.00025(00)	1024	29.53(0.22)	1.0956(25)
NB11-18.1	54.5(1.4)	n.a.	0.2616(12)	1.0862(17)	0.00050(01)	525	29.82(0.17)	1.0938(18)
NB11-21.2	56.5(1.5)	n.a.	0.2643(19)	1.0903(25)	0.00025(01)	1075	30.06(0.26)	1.0983(27)
NB11-15.3	65.0(1.5)	n.a.	0.3324(22)	1.0845(27)	0.00102(01)	325	39.55(0.34)	1.0945(30)
NB11-22.2	66.0(2.0)	n.a.	0.3268(22)	1.0789(25)	0.00070(01)	465	39.03(0.34)	1.0881(27)
NB11-19.1	67.0(1.0)	n.a.	0.3316(21)	1.0828(30)	0.00155(02)	214	39.46(0.33)	1.0926(33)
NB11-23.2	71.5(0.5)	n.a.	0.3601(19)	1.0951(20)	0.00431(04)	84	42.75(0.31)	1.1073(22)
NB11-16.3	71.5(1.5)	n.a.	0.3652(34)	1.0954(25)	0.01664(33)	22	42.34(0.63)	1.1075(28)
NB11-20.1	73.5(1.5)	n.a.	0.4449(38)	1.0778(26)	0.03713(58)	12	54.06(1.02)	1.0907(30)
NB11-24.2	75.5(1.5)	n.a.	0.4608(42)	1.0808(25)	0.01486(24)	31	58.66(0.82)	1.0954(29)
NB11-17.3	75.5(1.5)	n.a.	0.4591(22)	1.0769(27)	0.02245(41)	20	57.95(0.63)	1.0906(31)
NB11-9	78.0(2.0)	89	0.4815(28)	1.0834(29)	0.00381(04)	126	63.10(0.56)	1.0997(34)
NB11-25.2	79.0(0.9)	n.a.	0.4844(21)	1.0812(25)	0.00181(02)	268	64.00(0.44)	1.0973(30)
NB11-26.2	82.0(1.4)	n.a.	0.5339(27)	1.0782(26)	0.02224(19)	24	71.64(0.75)	1.0957(31)
NB11-21.1	83.0(0.9)	n.a.	0.5579(48)	1.0794(35)	0.02239(28)	25	76.29(1.14)	1.0985(43)
NB11-27.2	90.0(0.9)	n.a.	0.5725(37)	1.0774(27)	0.00131(02)	437	81.51(0.84)	1.0974(33)
NB11-22.1	92.5(1.5)	n.a.	0.5687(34)	1.0768(28)	0.00054(01)	1056	80.86(0.78)	1.0965(35)
NB11-23.1	97.5(1.5)	n.a.	0.5696(41)	1.0749(26)	0.00027(01)	2131	81.31(0.91)	1.0942(32)
NB11-28.2	104.0(0.9)	n.a.	0.5688(29)	1.0756(21)	0.00060(01)	943	81.02(0.66)	1.0950(26)
*NB11-24.1	107.0(1.0)	n.a.	0.5768(25)	1.0699(20)	0.02070(33)	28	81.54(0.73)	1.0880(25)
NB11-29.2	110.5(1.4)	n.a.	0.5763(29)	1.0705(24)	0.01606(16)	36	81.80(0.76)	1.0888(30)
NB11-5a	111.0(0.9)	53	0.5736(41)	1.0753(22)	0.00033(00)	1753	82.09(0.91)	1.0949(27)
NB11-25.1	114.0(0.9)	n.a.	0.5769(40)	1.0796(24)	0.01223(17)	47	81.15(0.93)	1.1001(30)
NB11-30.2	116.5(1.5)	n.a.	0.5829(30)	1.0695(32)	0.00650(06)	90	84.25(0.78)	1.0882(39)
NB11-10	136.5(0.5)	88	0.5975(29)	1.0720(27)	0.00254(03)	235	87.51(0.76)	1.0922(34)
NB11-18.3	142.5(1.5)	n.a.	0.5958(57)	1.0690(26)	0.00084(02)	713	87.70(1.33)	1.0884(32)
NB11-19.3	147.5(1.5)	n.a.	0.6004(38)	1.0694(22)	0.01235(10)	49	87.60(0.94)	1.0889(28)
NB11-20.3	167.5(1.5)	n.a.	0.5990(29)	1.0705(27)	0.00035(00)	1719	88.27(0.75)	1.0904(34)
NB11-31.2	172.0(1.0)	n.a.	0.6029(28)	1.0699(23)	0.00321(04)	188	88.96(0.72)	1.0899(29)
NB11-26.1	172.5(1.5)	n.a.	0.5999(22)	1.0711(16)	0.00106(01)	564	88.32(0.54)	1.0912(20)
NB11-32.2	177.0(0.5)	n.a.	0.6120(61)	1.0600(28)	0.00109(02)	562	92.72(1.51)	1.0780(35)
NB11-27.1	177.5(0.5)	n.a.	0.6140(45)	1.0594(33)	0.00326(06)	188	93.08(1.20)	1.0773(42)
NB11-33.2	180.5(0.5)	n.a.	0.6090(30)	1.0557(18)	0.00108(02)	565	92.67(0.77)	1.0724(23)
NB11-11	181.0(1.0)	89	0.6159(31)	1.0583(27)	0.00104(01)	594	93.92(0.85)	1.0760(34)
NB11-28.1	186.5(0.5)	n.a.	0.6405(31)	1.0640(18)	0.00734(11)	87	98.43(0.84)	1.0845(23)
*NB11-34.2	188.5(0.5)	n.a.	0.6541(36)	1.0566(24)	0.00275(03)	238	103.64(1.04)	1.0759(31)
NB11-29.1	189.5(0.5)	n.a.	0.6345(30)	1.0575(25)	0.00153(02)	415	98.57(0.86)	1.0759(32)
NB11-30.1	192.5(0.5)	n.a.	0.6487(42)	1.0518(23)	0.00380(05)	171	103.01(1.19)	1.0693(30)
NB11-35.2	193.5(0.5)	n.a.	0.6480(35)	1.0520(17)	0.00181(02)	358	102.97(0.97)	1.0695(22)
NB11-36.2	204.0(1.0)	n.a.	0.6500(28)	1.0490(21)	0.00212(02)	306	104.02(0.84)	1.0657(28)
NB11-2a	209.0(1.0)	55	0.6578(46)	1.0494(18)	0.00258(03)	255	106.00(1.30)	1.0666(24)
NB11-3a	215.0(1.0)	70	0.6727(44)	1.0541(19)	0.00145(02)	465	109.27(1.28)	1.0737(25)
NB11-37.2	215.5(1.5)	n.a.	0.6724(23)	1.0516(20)	0.00140(02)	481	109.69(0.75)	1.0703(26)
NB11-12	267.5(0.5)	103	0.6854(38)	1.0456(25)	0.00065(01)	1052	114.77(1.26)	1.0630(33)
NB11-6	332.0(1.0)	70	0.6898(30)	1.0385(24)	0.00498(05)	139	117.25(1.08)	1.0536(33)
NB11-4a	451.5(0.5)	46	0.7053(25)	1.0437(25)	0.00511(04)	138	120.81(1.01)	1.0614(34)
**NB 15-1**								
NB15_1.2_A	78.5(1.0)	202	0.6205(25)	0.9662(18)	0.00614(12)	101	112.24(0.98)	0.9536(26)
NB15_1.2_A2	99.5(0.0)	165	0.6590(27)	0.9882(19)	0.02511(50)	26	117.12(1.26)	0.9836(27)
NB15_1.2_B	117.5(1.0)	311	0.6258(24)	0.9500(17)	0.00262(05)	239	118.58(0.97)	0.9301(25)
NB15_1.2_C	157.0(1.0)	196	0.6513(26)	0.9770(18)	0.00648(13)	100	119.71(1.00)	0.9677(26)
NB15_1.2_D	183.0(1.0)	181	0.6537(27)	0.9781(18)	0.00830(17)	79	120.03(1.05)	0.9692(25)
NB15_1.2_E	205.0(1.0)	227	0.6566(25)	0.9767(17)	0.01385(28)	47	120.71(1.11)	0.9672(24)
NB15_1.2_F	219.0(1.0)	157	0.6810(26)	0.9891(18)	0.05244(105)	13	120.99(1.90)	0.9846(25)
NB15 1.4A	566.0(3.0)	145	0.6794(44)	0.9932(22)	0.00362(01)	188	125.37(1.63)	0.9902(31)
NB15 1.4 A6	606.0(1.0)	224	0.6803(17)	0.9936(26)	0.00182(04)	374	125.77(0.99)	0.9908(38)
*NB15 1.5 A	640.0(1.0)	110	0.7223(26)	0.9838(28)	0.14172(283)	5	117.72(31.08)	0.9775(45)
NB15 1.4B	735.5(2.5)	158	0.6793(28)	0.9908(24)	0.02017(07)	34	124.00(1.32)	0.9868(34)
NB15 1.5C	868.5(3.5)	257	0.6795(45)	0.9843(22)	0.00167(00)	406	128.23(1.75)	0.9775(32)
NB15 1.6D	1008.0(3.0)	161	0.6786(26)	0.9801(29)	0.00538(02)	126	128.64(1.32)	0.9714(44)
**NB15-2**								
NB15 2.1A	4.5(4.5)	58	0.0289(09)	1.2022(23)	0.02504(42)	1	0.46(0.47)	1.2025(23)
NB15 2.1B	63.0(3.0)	68	0.0493(11)	1.1951(25)	0.00339(06)	15	4.24(0.12)	1.1975(25)
NB15-2.1-B2	95.5(1.0)	84	0.0687(08)	1.1966(26)	0.00166(03)	41	6.13(0.24)	1.2035(26)
NB15-2.1-B3	137.5(1.0)	99	0.0912(08)	1.1982(25)	0.01177(24)	8	6.91(1.61)	1.2040(27)
NB15 2.1C	159.0(4.0)	76	0.0843(10)	1.2098(27)	0.00484(10)	17	7.39(0.13)	1.2142(28)
NB15-2.1-C2	193.5(1.0)	105	0.1039(10)	1.2004(26)	0.01510(30)	7	7.73(2.11)	1.2045(29)
*NB15 2.1D	225.5(3.5)	52	0.2917(24)	1.1691(32)	0.01508(22)	19	29.71(0.42)	1.1839(35)
NB15-2.1.D2	244.0(1.0)	62	0.5148(30)	1.1517(28)	0.04154(83)	12	57.82(6.16)	1.1766(44)
NB15 2.1E	290.5(3.5)	99	0.5294(37)	1.1252(21)	0.02670(58)	20	65.92(0.85)	1.1508(25)
**NB15-3**								
NB15 3.1 A (2016)	3.3(3.3)	54	0.0257(09)	1.1915(34)	0.02516(47)	1	-0.03(2.39)	1.1915(36)
NB15-3.1-A (2019)	4.5(1.0)	82	0.0104(03)	1.1916(26)	0.00565(11)	2	0.37(0.52)	1.1918(26)
NB15-3.1-D (2019)	58.0(1.0)	53	0.0526(08)	1.2433(28)	0.00378(08)	14	4.31(0.34)	1.2463(29)
NB15 3.1 B (2016)	89.0(4.0)	45	0.0707(07)	1.2183(39)	0.00948(10)	7	5.59(0.85)	1.2218(40)
NB15-3.1-F (2019)	94.5(1.0)	67	0.0774(08)	1.2293(26)	0.01573(31)	5	5.61(1.41)	1.2330(28)
NB15 3.2 A (2019)	109.0(1.5)	50	0.0890(10)	1.2346(37)	0.02591(52)	3	5.78(2.31)	1.2385(41)
NB15 3.2 C (2016)	157.0(5.0)	147	0.2312(13)	1.2247(31)	0.00469(08)	49	22.19(0.44)	1.2393(33)
NB15 3.2 D (2016)	232.0(3.5)	79	0.4489(23)	1.1733(33)	0.01404(16)	32	50.55(1.33)	1.1999(38)
NB15 3.2 D (2019)	272.0(1.5)	42	0.4684(30)	1.1565(37)	0.01686(34)	28	54.22(1.66)	1.1824(43)
NB15 3.2 E (2016)	335.0(5.5)	55	0.5306(30)	1.1738(37)	0.01526(46)	35	63.01(1.51)	1.2077(44)
**HC15-2**								
HC15-2A	1.9(1.9)	165	0.0194(02)	1.7306(95)	0.00233(03)	8	1.03(0.03)	1.7330(96)
HC15 2 U-A-1	13.6(1.0)	120	0.0567(08)	1.5479(43)	0.00109(02)	52	3.92(0.06)	1.5541(43)
HC15-2B	19.2(2.0)	138	0.0848(08)	1.5432(85)	0.00161(02)	53	5.97(0.07)	1.5525(86)
HC15 2 U-B-1	31.5(1.0)	180	0.1172(07)	1.6176(42)	0.00254(04)	46	7.94(0.06)	1.6317(43)
HC15 2 U-B-2	36.0(1.0)	172	0.1287(10)	1.6428(44)	0.00158(04)	82	8.69(0.08)	1.6589(45)
HC15 2 U-B-3	41.2(1.0)	234	0.1372(08)	1.6794(45)	0.00100(00)	137	9.12(0.06)	1.6972(46)
HC15 2 U-B-4	48.8(1.0)	423	0.1647(07)	1.8473(49)	0.00021(00)	797	10.04(0.05)	1.8718(50)
HC15-2C	54.0(2.5)	297	0.1829(10)	1.7991(100)	0.00070(01)	263	11.50(0.10)	1.8257(101)
HC15_2_VD_XX_A	84.3(4.5)	193	0.2203(12)	1.8562(28)	0.00128(03)	173	13.47(0.14)	1.8895(29)
**HC15-3**								
HC15-3UA	67.5(0.5)	453	0.8522(47)	1.5174(85)	0.00020(00)	4181	84.80(1.00)	1.6574(97)
HC15-3UB	178.0(1.0)	463	0.8779(44)	1.5510(89)	0.00019(00)	4525	85.56(0.97)	1.7017(99)

HC15-3UC	301.0(1.0)	383	0.8626(39)	1.5209(84)	0.00017(00)	5161	86.00(0.94)	1.6641(94)
HC15-3UD	351.0(1.0)	337	0.8597(39)	1.5219(86)	0.00015(00)	5580	85.49(0.93)	1.6645(95)
HC15-3UE	430.5(0.5)	361	0.9360(43)	1.6298(92)	0.00054(01)	1733	86.93(0.95)	1.8051(102)
HC15-3.11	440.0(10.0)	396	0.9133(46)	1.5908(37)	0.00113(00)	805	87.07(0.71)	1.7555(43)
**HC15-1**								
HC15-1.1-C	50.5(1.0)	163	0.1834(09)	1.4950(28)	0.00820(16)	22	13.23(0.90)	1.5087(31)
HC15-1.1-F	122.5(1.0)	271	0.1692(08)	1.4666(27)	0.00118(02)	143	13.04(0.15)	1.4877(27)
HC15-1.1-J	279.0(1.0)	137	0.1844(11)	1.3505(27)	0.00207(04)	89	15.57(0.27)	1.3658(28)
HC15-1.1-K	289.0(1.0)	255	0.3850(14)	1.2949(24)	0.02849(57)	14	34.43(3.69)	1.3196(42)
HC15-1.1-F	122.5(1.0)	271	0.1692(08)	1.4666(27)	0.00118(02)	143	13.04(0.15)	1.4877(27)
HC15-1.1-J	279.0(1.0)	137	0.1844(11)	1.3505(27)	0.00207(04)	89	15.57(0.27)	1.3658(28)
HC15-1.1-K	289.0(1.0)	255	0.3850(14)	1.2949(24)	0.02849(57)	14	34.43(3.69)	1.3196(42)

[a] Activity ratios determined after Hellstrom (2003) and Drysdale et al (2012)

[b] Age in kyr before 1950 AD corrected for intial ^{230}Th using eqn. 1 of Hellstrom (2006), the decay constants of Cheng et al (2013) and [^{230}Th/^{232}Th]$_i$ of 0.43 ± 0.043

[c] Initial [^{234}U/^{238}U] calculated using corrected age

* Outlier not used for age-depth model

2-σ uncertainties in brackets are of the last two significant figures presented

Cheng, H., Lawrence Edwards, R., Shen, C.-C., Polyak, V.J., Asmerom, Y., Woodhead, J.D., Hellstrom, J., Wang, Y., Kong, X., Spötl, C., Wang, X., Calvin Alexander, E., Jr, 2013. Improvements in 230Th dating, 230Th and 234U half-life values, and U–Th isotopic measurements by multi-collector inductively coupled plasma mass spectrometry. Earth and Planetary Science Letters 371–372, 82–91.

Drysdale, R.N., Paul, B.T., Hellstrom, J., Couchoud, I., Greig, A., Bajo, P., Zanchetta, G., Isola, I., Spötl, C., Baneschi, I., Regattieri, E., Woodhead, J.D., 2012. Precise microsampling of poorly laminated speleothems for U-series dating. Quaternary Geochronology 14, 38–47.

Hellstrom, J., 2006. U–Th dating of speleothems with high initial 230Th using stratigraphical constraint. Quaternary Geochronology 1, 289–295.

Hellstrom, J., 2003. Rapid and accurate U/Th dating using parallel ion-counting multi-collector ICP-MS. Journal of Analytical Atomic Spectrometry 18, 1346–1351.

Supplementary table 2. Relative growth probability curves vs time for Mt Arthur speleothems

AgeKa	MD3	ED1	NB3	NB5	NB6	NB7	NB8	NB9	NB11	NB15-1	NB15-2	NB15-3	HC15-1	HC15-2	HC15-3
0.25	0.46387222	0.0007	0.001155	0.326933	0.00400056	0.008265	0.00019231	0	0.01148	0	0.2916125	0.681285	0.0000048	0.141012	0
0.75	0.50097778	0.003325	0.00132417	0.79128667	0.00840434	0.11817	0.00060769	0	0.002864	0	0.29308542	0.680215	0.0000032	0.243171	0
1.25	0.50064444	0.15757	0.00180833	0.77563067	0.02642269	0.54321	0.00063846	0	0.004876	0	0.29134792	0.67465	0.0000032	0.358965	0
1.75	0.45181111	0.907018	0.00170333	0.77083	0.06409597	1.05996	0.00084231	0	0.011784	0	0.2926625	0.683515	0.000004	0.355338	0
2.25	0.383075	1.217244	0.00204167	0.772654	0.16475834	1.2578775	0.00100385	0	0.087088	0	0.29337708	0.68088	0.000004	0.356958	0
2.75	0.38158611	1.353016	0.00258417	0.76165933	0.32066169	1.27788	0.00133462	0	0.066788	0	0.29317708	0.68309	0.0000032	0.355914	0
3.25	0.38471111	1.340178	0.00299833	0.54471733	0.51297331	1.2812775	0.00177308	0	0.008916	0	0.29412292	0.672915	0.0000048	0.35829	0
3.75	0.37871667	1.345127	0.00331917	0.53640167	0.68152666	1.27971	0.00245385	0	0.005412	0	0.29988542	0.683035	0.000004	0.350361	0
4.25	0.3743	1.344406	0.00394917	0.53358333	0.83505022	1.2813	0.00367308	0	0.005588	0	0.33189167	1.02221	0.0000056	0.249624	0
4.75	0.43073889	1.342012	0.00442167	0.534052	0.93447922	1.277985	0.00590769	0	0.005576	0	0.33032083	1.16574	0.0000096	0.2439	0
5.25	0.76286111	1.239448	0.00550667	0.534489	0.981387	1.280265	0.01239615	0	0.00522	0	0.33269375	1.3107	0.0000136	0.247275	0
5.75	0.76004722	0.90118	0.005985	0.539885	0.99825894	1.25376	0.0585	0	0.005016	0	0.34361875	1.8408	0.0000208	0.282771	0
6.25	1.35721111	0.890162	0.00685417	0.53840933	0.9855615	1.063605	0.18634231	0	0.005268	0	0.78058958	0.187405	0.000028	0.560655	0
6.75	1.55816667	0.883519	0.0078575	0.535705	0.88522328	0.9728025	0.06406923	0	0.005288	0	0.81145	0.152335	0.0000296	0.568098	0
7.25	1.28549722	0.882364	0.00851083	0.53763033	0.79687094	0.9604425	0.01265385	0	0.005432	0	1.60174167	0.15046	0.000036	0.567	0
7.75	1.27469722	0.92918	0.01007417	0.5472	0.72475803	0.95565	0.00603846	0	0.005096	0	0.79421875	0.14969	0.0000656	0.543564	0
8.25	1.11730833	1.162063	0.01113	0.88899733	0.69788469	0.960135	0.00374231	0	0.005472	0	0.70526042	0.148505	0.000112	0.527175	0
8.75	1.08825833	1.225392	0.01257083	0.90963767	0.67828509	0.96252	0.00272308	0	0.005332	0	0.37642292	0.147925	0.0001968	0.828459	0
9.25	0.89643889	1.228759	0.01428583	0.90659133	0.65378363	0.965295	0.00182692	0	0.005192	0	0.1511125	0.147	0.0002872	0.813555	0
9.75	0.81864444	1.159648	0.01535917	0.904647	0.631741	0.966855	0.00141923	0	0.005428	0	0.06864583	0.14707	0.0005184	0.717255	0
10.25	0.19030556	1.18181	0.017255	0.899878	0.59825803	0.96354	0.00106154	0	0.00508	0	0.03682708	0.14657	0.0010024	0.39672	0
10.75	0.47353333	1.532433	0.01792	0.89357	0.56495691	0.969675	0.00099231	0	0.005336	0	0.02048333	0.14698	0.0025608	0.318726	0
11.25	0.82128056	1.696464	0.0197575	0.89771833	0.53305519	0.985725	0.00076154	0	0.00534	0	0.01245833	0.14741	0.0084712	0.33642	0
11.75	1.00310556	0.012117	0.02079	0.929651	0.50164366	0.5406075	0.00059615	0	0.005324	0	0.00814167	0.14656	0.0325672	1.26036	0
12.25	0.50068611	0.005593	0.021525	1.27760433	0.4532495	0.04845	0.00056539	0	0.00504	0	0.00566042	0.146765	0.1435488	1.346463	0
12.75	0.42667778	0.006608	0.02307083	1.27370933	0.39887822	0.0090075	0.00046154	0	0.00516	0	0.00386875	0.14686	1.641304	1.349883	0
13.25	0.60941944	0.003262	0.0239225	1.28094833	0.34997016	0.003075	0.00048462	0	0.005168	0	0.00279583	0.147685	0.598816	1.373508	0
13.75	0.74399167	0.003241	0.02485583	1.29379867	0.30027147	0.00117	0.00035769	0	0.005316	0	0.00201667	0.147605	0.4798528	0.779958	0
14.25	1.253475	0.00343	0.0251475	1.28006167	0.24426359	0.0005325	0.00031923	0	0.00524	0	0.00148958	0.14749	0.4800904	0.127512	0
14.75	0.55020556	0.002982	0.02587083	0.48447467	0.19235116	0.000285	0.00021923	0	0.00494	0	0.00116458	0.1481	0.4786064	0.033615	0
15.25	0.403625	0.00336	0.02659417	0.20376233	0.14595728	0.0001575	0.00024231	0	0.005328	0	0.0008625	0.149135	0.450632	0.012555	0
15.75	0.42093889	0.003045	0.02671083	0.202806	0.10827609	0.0000975	0.00021539	0	0.005332	0	0.00072917	0.149	0.0938144	0.005706	0
16.25	0.28676944	0.003227	0.02732333	0.20158367	0.07781331	0.000075	0.00023077	0	0.005	0	0.0005375	0.148235	0.0029664	0.00324	0
16.75	0.23836944	0.003297	0.02790083	0.19967733	0.05312209	0.0000525	0.00012308	0	0.005284	0	0.00043125	0.14819	0.0004584	0.001899	0
17.25	0.18027222	0.003269	0.02765583	0.196916	0.035926	0.00003	0.00011539	0	0.005228	0	0.00036875	0.14746	0.0001552	0.000963	0
17.75	0.17595	0.002863	0.02795333	0.19810667	0.02205053	0.0000375	0.00017308	0	0.005456	0	0.0002625	0.1477	0.0000664	0.000675	0
18.25	0.05289722	0.003073	0.02777833	0.198683	0.01514047	0.0000225	0.00013846	0	0.005084	0	0.00024375	0.14767	0.0000328	0.000504	0
18.75	0.04826389	0.003038	0.02888083	0.19993067	0.00992234	0	0.00011539	0	0.005188	0	0.00018958	0.147155	0.0000264	0.000351	0
19.25	0.04714167	0.003269	0.02741083	0.20030433	0.00669659	0.000015	9.62E-05	0	0.00526	0	0.00013542	0.148015	0.0000328	0.000216	0
19.75	0.04786944	0.003269	0.02837917	0.19994333	0.003795	0.0000225	0.00011923	0	0.005408	0	0.00012708	0.14866	0.000028	0.000207	0
20.25	0.04753889	0.003101	0.02828	0.19981033	0.00207934	0	0.00010769	0	0.005628	0	0.00010417	0.148825	0.000028	0.000153	0
20.75	0.04764722	0.00315	0.02814583	0.20065267	0.00087759	0.0000075	0.00012308	0	0.006628	0	9.79E-05	0.14953	0.000028	0.000117	0
21.25	0.04668889	0.003304	0.02764417	0.20302133	0.00043484	0.000015	9.23E-05	0	0.039956	0	8.96E-05	0.15006	0.0000272	0.000126	0
21.75	0.04636389	0.003017	0.0287175	0.20466167	0.00023719	0	0.0001	0	0.0499	0	6.46E-05	0.15023	0.0000344	0.000126	0
22.25	0.04605833	0.002814	0.02776083	0.20418033	9.49E-05	0	0.00010769	0	0.049928	0	7.08E-05	0.13659	0.0000344	0.000099	0
22.75	0.04658889	0.003003	0.02823333	0.20404733	6.33E-05	0.0000225	8.08E-05	0	0.054276	0	0.00005	0.13532	0.0000472	0.000081	0
23.25	0.04691111	0.002968	0.028315	0.20254633	4.74E-05	0	8.08E-05	0	0.178012	0	0.0000625	0.13427	0.0000472	0.00009	0
23.75	0.05343056	0.003073	0.0281925	0.19975967	3.16E-05	0	6.92E-05	0	0.127872	0	4.79E-05	0.13391	0.0000456	0.000054	0
24.25	0.11333889	0.003038	0.02811667	0.19949367	3.95E-05	0.000015	6.92E-05	0	0.1596	0	0.00005	0.13385	0.0000528	0.000063	0
24.75	0.11553333	0.002989	0.0278425	0.20039933	1.58E-05	0	7.69E-05	0	0.199908	0	4.58E-05	0.13312	0.0000672	0.000054	0
25.25	0.11457222	0.003031	0.02785417	0.201913	1.58E-05	0	6.92E-05	0	0.200804	0	4.58E-05	0.1336	0.0000632	0.000027	0
25.75	0.11560556	0.002975	0.02821	0.20169767	3.95E-05	0	6.54E-05	0	0.333188	0	4.58E-05	0.132385	0.0000704	0.000054	0
26.25	0.11483056	0.003248	0.02798833	0.20310367	0	0	0.00005	0	0.235604	0	5.21E-05	0.132245	0.0000808	0.000009	0
26.75	0.11518611	0.003339	0.0277725	0.20357867	1.58E-05	0	3.85E-05	0	0.423256	0	3.96E-05	0.132045	0.0001096	0.000036	0
27.25	0.11641667	0.003087	0.02825083	0.191995	7.91E-06	0	7.31E-05	0	0.350388	0	4.79E-05	0.13165	0.00012	0.000027	0
27.75	0.11895556	0.003108	0.02746333	0.11174533	0	0	7.31E-05	0	0.11642	0	4.58E-05	0.132715	0.0001392	0.000018	0
28.25	0.22715833	0.003185	0.02783083	0.109117	2.37E-05	0	0.00005	0	0.148092	0	5.42E-05	0.13245	0.0001648	0.000009	0
28.75	0.32263611	0.003045	0.02741083	0.108034	7.91E-06	0	1.54E-05	0	0.4793	0	3.54E-05	0.13224	0.000208	0.000018	0
29.25	0.32335833	0.002842	0.02832667	0.10817333	0	0	0.00005	0	0.466244	0	0.00005	0.13161	0.0002928	0.000009	0
29.75	0.36064444	0.003031	0.02737	0.10795167	0	0	4.62E-05	0	0.491896	0	5.21E-05	0.131455	0.0003568	0.000009	0
30.25	0.38594167	0.003136	0.02807	0.10753367	0	0	4.62E-05	0	0.108236	0	4.58E-05	0.132205	0.0004832	0	0
30.75	0.38428611	0.003178	0.02750417	0.10694467	0	0	2.31E-05	0	0.037344	0	5.42E-05	0.131675	0.0009352	0	0
31.25	0.39308056	0.003052	0.02748083	0.107046	0	0	0.00005	0	0.036964	0	0.0000625	0.131845	0.0012776	0	0
31.75	0.14708611	0.003234	0.027825	0.10642533	0	0	4.23E-05	0	0.036584	0	0.00004375	0.131855	0.0024984	0	0
32.25	0.06291944	0.003038	0.02767333	0.10524733	0	0	3.08E-05	0	0.035948	0	6.04E-05	0.132155	0.0037184	0	0
32.75	0.03517222	0.002968	0.02781333	0.10553233	0	0	5.77E-05	0	0.03658	0	7.08E-05	0.132275	0.005688	0	0
33.25	0.02191111	0.003374	0.02729417	0.105336	7.91E-06	0	3.08E-05	0	0.036532	0	0.0000625	0.132015	0.008356	0	0
33.75	0.01505278	0.00294	0.02748083	0.105374	0	0	3.46E-05	0	0.036272	0	8.33E-05	0.1314	0.0097256	0	0
34.25	0.01066389	0.003206	0.02744583	0.10494333	0	0	3.08E-05	0	0.036	0	8.33E-05	0.131655	0.0097296	0	0
34.75	0.00803333	0.002961	0.0277025	0.104861	0	0	2.31E-05	0	0.036024	0	8.54E-05	0.131215	0.0090936	0	0
35.25	0.00615278	0.003017	0.0273875	0.105526	0	0	2.31E-05	0	0.036324	0	0.0001125	0.131035	0.0068752	0	0
35.75	0.004925	0.003157	0.02788917	0.10515233	0	0	2.31E-05	0	0.035952	0	0.00011875	0.130845	0.0047936	0	0
36.25	0.00406667	0.003087	0.02745167	0.10519667	0	0	3.46E-05	0	0.035776	0	0.00012083	0.13113	0.0035816	0	0
36.75	0.00335833	0.003059	0.02776667	0.10433533	0	0	2.31E-05	0	0.035796	0	0.00013958	0.130745	0.0025616	0	0
37.25	0.00286389	0.00315	0.02756833	0.104538	7.91E-06	0	2.31E-05	0	0.03604	0	0.0001375	0.130915	0.0017704	0	0
37.75	0.00245833	0.003129	0.02724167	0.10496233	0	0	2.31E-05	0	0.03618	0	0.00014792	0.13129	0.0009384	0	0
38.25	0.00208333	0.002975	0.02765	0.10431	0	0	3.08E-05	0	0.036608	0	0.00020833	0.130755	0.0006688	0	0
38.75	0.00188333	0.002975	0.02765583	0.104861	7.91E-06	0	2.69E-05	0	0.037088	0	0.00020833	0.13098	0.0005152	0	0
39.25	0.00158611	0.003017	0.02779	0.10479767	7.91E-06	0	1.54E-05	0	0.16208	0	0.00022917	0.13055	0.0004272	0	0
39.75	0.00131944	0.003416	0.027615	0.10515867	0	0	3.85E-05	0	0.0884	0	0.00022917	0.129875	0.0003576	0	0
40.25	0.00129722	0.003094	0.02804083	0.10532967	0	0	3.85E-05	0	0.054324	0	0.00028542	0.130285	0.0002872	0	0
40.75	0.0011	0.003262	0.0273	0.10555133	7.91E-06	0	2.31E-05	0	0.054552	0	0.00028333	0.130355	0.0002504	0	0
41.25	0.000975	0.003052	0.028105	0.105051	0	0	1.15E-05	0	0.05426	0	0.00035625	0.131335	0.0002424	0	0
41.75	0.00093889	0.003045	0.02783083	0.104937	0	0	3.46E-05	0	0.054036	0	0.00035	0.131105	0.000188	0	0
42.25	0.00076944	0.003087	0.02763833	0.10572233	0	0	1.92E-05	0	0.053092	0	0.00040833	0.130775	0.000172	0	0
42.75	0.0008	0.003164	0.02821	0.105355	0	0	3.46E-05	0	0.05584	0	0.00045208	0.13162	0.0001376	0	0
43.25	0.00063333	0.003108	0.02776667	0.10538667	7.91E-06	0	2.31E-05	0	0.007632	0	0.00048542	0.13116	0.0001432	0	0
43.75	0.00066389	0.003045	0.028	0.10542467	0	0	3.85E-06	0	0.00648	0	0.00053958	0.130405	0.0001288	0	0
44.25	0.00061667	0.003059	0.02790667	0.10523467	0	0	2.31E-05	0	0.00636	0	0.0006125	0.13063	0.0001072	0	0
44.75	0.000525	0.003101	0.02822167	0.105944	7.91E-06	0	2.31E-05	0	0.00616	0	0.0006875	0.130695	0.0001056	0	0
45.25	0.0005	0.003122	0.02763833	0.10569067	7.91E-06	0	1.54E-05	0	0.006384	0	0.0008125	0.130705	0.0000952	0	0
45.75	0.00044722	0.002856	0.028105	0.105469	7.91E-06	0	1.15E-05	0	0.00664	0	0.00094167	0.131015	0.0000824	0	0
46.25	0.00045556	0.003017	0.027895	0.10511433	7.91E-06	0	2.31E-05	0	0.006188	0	0.00107292	0.13081	0.0000744	0	0
46.75	0.00038056	0.003101	0.02799417	0.10521567	7.91E-06	0	2.31E-05	0	0.006084	0	0.0012125	0.131395	0.0000688	0	0
47.25	0.00038333	0.002968	0.02783083	0.105089	7.91E-06	0	3.46E-05	0	0.005952	0	0.001375	0.13107	0.0000608	0	0
47.75	0.00033889	0.003143	0.0281925	0.10462667	7.91E-06	0	2.69E-05	0	0.0059	0	0.00151667	0.132445	0.0000496	0	0
48.25	0.00035278	0.003115	0.02759167	0.104196	1.58E-05	0	2.69E-05	0	0.006208	0	0.00177917	0.132345	0.0000456	0	0
48.75	0.00029722	0.002975	0.02860667	0.103854	0	0	1.15E-05	0	0.006192	0	0.002025	0.132985	0.0000488	0	0
49.25	0.00029167	0.003213	0.0278075	0.104082	1.58E-05	0	1.15E-05	0	0.00614	0	0.00237708	0.133405	0.0000456	0	0
49.75	0.00029444	0.002912	0.02791833	0.10361967	7.91E-06	0	2.31E-05	0	0.006192	0	0.00273958	0.134675	0.0000376	0	0

50.25	0.00023889	0.003227	0.028245	0.10363867	3.16E-05	0	1.92E-05	0	0.00598	0	0.00322083	0.198135	0.0000376	0	0
50.75	0.00022222	0.003136	0.02821583	0.10331567	1.58E-05	0	1.15E-05	0	0.006044	0	0.0038125	0.46963	0.0000264	0	0
51.25	0.00025278	0.003241	0.02814583	0.10409467	7.91E-06	0	1.15E-05	0	0.005912	0	0.00447708	0.54045	0.0000408	0	0
51.75	0.00026389	0.003136	0.0282625	0.10348667	0	0	1.15E-05	0	0.006088	0	0.00545833	0.541485	0.0000264	0	0
52.25	0.00018611	0.003164	0.02851333	0.10405033	1.58E-05	0	1.54E-05	0	0.006152	0	0.00648333	0.54499	0.000032	0	0
52.75	0.00023889	0.002975	0.02829167	0.10428467	1.58E-05	0	1.54E-05	0	0.006056	0	0.00815208	0.54559	0.0000304	0	0
53.25	0.00017222	0.003241	0.02793	0.105222	0	0	1.54E-05	0	0.006068	0	0.00982708	0.54263	0.0000264	0	0
53.75	0.0002	0.002758	0.0281575	0.10467733	2.37E-05	0	1.15E-05	0	0.008228	0	0.01234375	0.52773	0.0000216	0	0
54.25	0.00017222	0.003164	0.02825083	0.10511433	1.58E-05	0	3.85E-06	0	0.011376	0	0.01556875	0.432055	0.0000224	0	0
54.75	0.00014722	0.003003	0.02780167	0.10527267	1.58E-05	0	1.92E-05	0	0.014216	0	0.01968958	0.37341	0.0000208	0	0
55.25	0.00015833	0.003248	0.02821	0.10564633	1.58E-05	0	2.69E-05	0	0.015856	0	0.02605625	0.36438	0.0000248	0	0
55.75	0.00018333	0.003052	0.0282275	0.106096	1.58E-05	0	3.85E-06	0	0.015932	0	0.03477917	0.360935	0.0000136	0	0
56.25	0.00016111	0.003052	0.02802917	0.106001	5.53E-05	0	1.15E-05	0	0.01572	0	0.04720833	0.360355	0.0000248	0	0
56.75	0.00010833	0.00329	0.02792417	0.106799	1.58E-05	0	1.92E-05	0	0.01676	0	0.0649875	0.36037	0.0000176	0	0
57.25	0.00016944	0.003052	0.02783083	0.10653933	3.16E-05	0	1.15E-05	0	0.018192	0	0.08940417	0.35953	0.000016	0	0
57.75	0.00010556	0.002961	0.02751583	0.10695733	3.16E-05	0	2.69E-05	0	0.021804	0	0.115475	0.359595	0.0000128	0	0
58.25	0.000125	0.0028	0.02809917	0.11262567	2.37E-05	0	1.54E-05	0	0.0441	0	0.13932083	0.360525	0.0000176	0	0
58.75	0.00013611	0.003115	0.02807583	0.14112567	1.58E-05	0	1.54E-05	0	0.026744	0	0.15203333	0.360205	0.0000144	0	0
59.25	0.00011667	0.003038	0.0278425	0.15180367	2.37E-05	0	2.69E-05	0	0.017728	0	0.1566125	0.360105	0.0000152	0	0
59.75	0.00010556	0.003465	0.02800583	0.151411	1.58E-05	0	7.69E-06	0	0.017252	0	0.15578542	0.356335	0.000016	0	0
60.25	0.00010556	0.00301	0.02814583	0.15104367	4.74E-05	0	2.69E-05	0	0.0172	0	0.15465625	0.357115	0.000012	0	0
60.75	0.000125	0.003269	0.02795917	0.151658	5.53E-05	0	7.69E-06	0	0.018168	0	0.154725	0.358605	0.0000144	0	0
61.25	9.17E-05	0.002751	0.02855417	0.150385	4.74E-05	0	7.69E-06	0	0.01796	0	0.15471458	0.361285	0.0000072	0	0
61.75	0.00011667	0.003486	0.02755667	0.15011267	5.53E-05	0	3.85E-06	0	0.018216	0	0.15554792	0.3658	0.0000128	0	0
62.25	8.61E-05	0.002821	0.028105	0.14916267	5.53E-05	0	1.15E-05	0	0.017776	0	0.15675208	0.367935	0.0000128	0	0
62.75	8.61E-05	0.003164	0.0284725	0.148941	5.53E-05	0	1.54E-05	0	0.019668	0	0.15623333	0.36944	0.000008	0	0
63.25	9.72E-05	0.003059	0.02783083	0.147877	0.00010278	0	7.69E-06	0	0.035228	0	0.15534792	0.37033	0.000012	0	0
63.75	6.67E-05	0.003311	0.02809917	0.148295	7.12E-05	0	1.15E-05	0	0.053552	0	0.155475	0.36953	0.0000064	0	0
64.25	0.00010833	0.002975	0.02820417	0.147269	7.12E-05	0	1.15E-05	0	0.021868	0	0.15663958	0.365915	0.0000048	0	0
64.75	8.06E-05	0.00343	0.02816917	0.14760467	6.33E-05	0	1.54E-05	0	0.014452	0	0.15217083	0.356995	0.0000112	0	0
65.25	6.11E-05	0.003332	0.028315	0.14737033	0.00013441	0	7.69E-06	0	0.014388	0	0.12714792	0.342285	0.0000072	0	0
65.75	8.06E-05	0.003339	0.02772	0.14780733	0.00010278	0	1.54E-05	0	0.014292	0	0.0780125	0.319755	0.0000104	0	0
66.25	0.00010278	0.003017	0.02804083	0.149093	0.00010278	0	1.92E-05	0	0.014428	0	0.03552083	0.291035	0.0000104	0	0
66.75	5.56E-05	0.003073	0.02830917	0.148637	0.00011859	0	3.85E-06	0	0.014724	0	0.01430417	0.25872	0.0000064	0	0
67.25	8.61E-05	0.003192	0.0279125	0.148428	0.00018184	0	1.15E-05	0	0.014368	0	0.00627917	0.22744	0.0000064	0	0
67.75	7.22E-05	0.00315	0.02790083	0.148713	0.00022928	0	1.54E-05	0	0.014772	0	0.00334583	0.19874	0.0000048	0	0
68.25	6.67E-05	0.003206	0.02809333	0.14885233	0.00022138	0	1.15E-05	0	0.014604	0	0.0018125	0.17461	0.0000104	0	0
68.75	0.000075	0.003206	0.0278075	0.14914367	0.00024509	0	3.85E-06	0	0.01446	0	0.00122083	0.15164	0.0000056	0	0
69.25	4.44E-05	0.003136	0.02781917	0.149435	0.00030044	0	7.69E-06	0	0.01474	0	0.00069167	0.133685	0.0000056	0	0
69.75	6.11E-05	0.003325	0.02811667	0.15016967	0.00034788	0	7.69E-06	0	0.014292	0	0.00046458	0.117315	0.0000104	0	0
70.25	0.00005	0.0035	0.02784833	0.14934633	0.0003795	0	1.15E-05	0	0.014508	0	0.00034375	0.104095	0.0000032	0	0
70.75	6.39E-05	0.005173	0.027405	0.149359	0.00042694	0	1.54E-05	0	0.01458	0	0.00024375	0.091885	0.000004	0	0
71.25	6.11E-05	0.013475	0.02835583	0.148428	0.00068784	0	1.54E-05	0	0.013976	0	0.00015	0.08163	0.0000096	0	0
71.75	6.11E-05	0.040929	0.02779	0.14844067	0.00066413	0	3.85E-06	0	0.011532	0	0.00012292	0.07315	0.0000072	0	0
72.25	3.89E-05	0.087724	0.02821	0.147953	0.00067994	0	1.15E-05	0	0.011828	0	9.58E-05	0.065645	0.0000072	0	0
72.75	4.44E-05	0.134806	0.02723	0.14853567	0.00094875	0	0	0	0.012504	0	0.0000625	0.05874	0.0000064	0	0
73.25	3.06E-05	0.158333	0.02786	0.14816833	0.00117013	0	0	0	0.014576	0	5.42E-05	0.05309	0.0000072	0	0
73.75	5.83E-05	0.166327	0.02808167	0.14800367	0.00121756	0	1.15E-05	0	0.014112	0	4.58E-05	0.04802	0.0000072	0	0
74.25	6.11E-05	0.164871	0.027615	0.14763	0.00193703	0	7.69E-06	0	0.013548	0	3.54E-05	0.043255	0.0000048	0	0
74.75	0.00005	0.165249	0.02786	0.14820633	0.00210306	0	7.69E-06	0	0.013404	0	2.92E-05	0.03897	0.0000024	0	0
75.25	4.72E-05	0.163989	0.02771417	0.14864333	0.00300438	0	1.15E-05	0	0.014452	0	0.0000375	0.035855	0.0000056	0	0
75.75	3.61E-05	0.163891	0.02819833	0.14786433	0.00344713	0	1.15E-05	0	0.018348	0	0.000025	0.032515	0.0000088	0	0
76.25	0.000025	0.180698	0.0275625	0.14678133	0.00454609	0	1.15E-05	0	0.029568	0	1.04E-05	0.02996	0.0000072	0	0
76.75	4.72E-05	0.19488	0.02794167	0.146604	0.00582691	0	1.15E-05	0	0.04406	0	2.71E-05	0.027305	0.0000056	0	0
77.25	2.78E-05	0.1729	0.02764417	0.14683833	0.00825413	0	2.31E-05	0	0.05278	0	0.00001875	0.024985	0.0000016	0	0
77.75	3.61E-05	0.18291	0.02771417	0.146794	0.01173288	0	1.54E-05	0	0.055352	0	0.0000125	0.023045	0.0000056	0	0
78.25	4.72E-05	0.183428	0.02772583	0.14811133	0.01704588	0	7.69E-06	0	0.056104	0	4.17E-06	0.021335	0.0000016	0	0
78.75	3.89E-05	0.171472	0.02713667	0.147915	0.02637525	0	0	0	0.056168	0	0.0000125	0.01961	0.0000024	0	0
79.25	0.000025	0.139041	0.02807583	0.14893467	0.041239	0	1.15E-05	0	0.055532	0	0.00000625	0.01854	0.0000024	0	0
79.75	2.22E-05	0.112252	0.02735833	0.14872567	0.06032469	0	3.85E-06	0	0.055716	0	0.0000125	0.017095	0.0000056	0	0
80.25	5.83E-05	0.087409	0.02737	0.149131	0.08365603	0	7.69E-06	0	0.057044	0	0.0000125	0.01616	0.0000056	0	0
80.75	0.000025	0.076573	0.02779583	0.15051167	0.103983	0	1.15E-05	0	0.249776	0	2.08E-06	0.01499	0.0000048	0	0
81.25	3.61E-05	0.073535	0.02766167	0.15025833	0.11548659	0	7.69E-06	0	1.093496	0	4.17E-06	0.01386	0.0000024	0	0
81.75	3.61E-05	0.07371	0.027545	0.16304533	0.1208075	0	7.69E-06	0	0.565752	0	8.33E-06	0.012825	0.0000056	0	0
82.25	2.78E-05	0.074284	0.02723	0.22183767	0.12218319	0	3.85E-06	0	0.118932	0	0.00000625	0.011895	0.0000048	0	0
82.75	1.94E-05	0.081886	0.0271075	0.53788367	0.12260222	0	0	0	0.043504	0	4.17E-06	0.01101	0.0000032	0	0
83.25	3.06E-05	0.098588	0.02797083	0.97435167	0.12242828	0	1.54E-05	0	0.043296	0	4.17E-06	0.01057	0.000004	0	0.00029284
83.75	2.22E-05	0.140903	0.0275975	1.42094667	0.12092609	0	7.69E-06	0	0.056996	0	4.17E-06	0.009765	0.0000056	0	0.00463951
84.25	2.78E-05	0.227703	0.02708417	1.248946	0.12244409	0	1.54E-05	0	0.121136	0	4.17E-06	0.009105	0.0000016	0	0.05683012
84.75	4.17E-05	0.533512	0.02744583	1.01791867	0.12189066	0	7.69E-06	0	0.218288	0	0	0.00857	0.0000032	0	0.29957926
85.25	3.61E-05	1.723918	0.02797083	0.97012733	0.12106841	0	1.92E-05	0	0.253516	0	1.04E-05	0.008155	0.0000056	0	0.88329436
85.75	8.33E-06	1.931811	0.02688583	0.98607467	0.12119491	0	3.85E-06	0	0.255936	0	2.08E-06	0.007625	0.0000032	0	1.31616148
86.25	3.61E-05	0.558257	0.02751583	1.00208533	0.12065728	0	1.92E-05	0	0.256356	0	2.08E-06	0.00728	0.0000008	0	0.61575357
86.75	2.22E-05	0.0196	0.02746333	1.00244	0.12018291	0	2.31E-05	0	0.265712	0	2.08E-06	0.00692	0.000004	0	0.30064049
87.25	3.33E-05	0.000154	0.02730583	0.90838367	0.11965319	0	2.69E-05	0	0.467248	0	2.08E-06	0.00663	0.0000056	0	0.12790913
87.75	2.22E-05	0.000042	0.02755667	0.612636	0.11999316	0	7.69E-06	0	1.165536	0	0	0.00619	0.0000048	0	0.02350864
88.25	1.67E-05	0	0.02783667	0.46860967	0.11943181	0	3.85E-06	0	1.188892	0	4.17E-06	0.006005	0.0000016	0	0.00588593
88.75	3.06E-05	0	0.02713083	0.45126267	0.11902859	0	1.54E-05	0	0.307748	0	2.08E-06	0.005555	0.0000016	0	0.00318074
89.25	1.94E-05	0	0.0273525	0.45439767	0.11875978	0	7.69E-06	0	0.048876	0	0	0.005225	0.000004	0	0.00195407
89.75	2.22E-05	0	0.02772	0.450832	0.11965319	0	1.15E-05	0	0.046108	0	0	0.004905	0.0000032	0	0.00132938
90.25	1.94E-05	0	0.02732333	0.45160467	0.11848306	0	0	0	0.047088	0	0	0.00467	0.0000056	0	0.00097679
90.75	1.39E-05	0	0.02758	0.45289033	0.11986666	0	0	0	0.046328	0	0.00000625	0.004455	0.0000024	0	0.00071309
91.25	1.67E-05	0	0.027335	0.45228233	0.11856213	0	3.85E-06	0	0.046088	0	0	0.00421	0.0000016	0	0.00054272
91.75	2.22E-05	0	0.02766167	0.50771167	0.11898116	0	1.54E-05	0	0.043464	0	0	0.003825	0.0000016	0	0.0004484
92.25	2.78E-05	0	0.02776667	0.74766267	0.11971644	0	1.92E-05	0	0.060396	0	2.08E-06	0.003835	0.0000008	0	0.00036889
92.75	1.94E-05	0	0.02732917	0.89101767	0.11868072	0	1.54E-05	0	0.152608	0	0	0.003595	0.0000008	0	0.00030667
93.25	2.22E-05	0	0.0279825	0.894121	0.11869653	0	1.54E-05	0	0.128748	0	0	0.003325	0.0000032	0	0.00025482
93.75	1.94E-05	0	0.02720083	0.88648933	0.11929741	0	1.15E-05	0	0.050472	0	2.08E-06	0.00335	0.0000024	0	0.00022963
94.25	1.39E-05	0	0.0276675	0.88453867	0.11875188	0	7.69E-06	0	0.040564	0	0	0.00306	0.0000024	0	0.0001921
94.75	2.78E-05	0	0.02763833	0.81902033	0.11946344	0	3.85E-06	0	0.04652	0	0	0.002955	0.0000048	0	0.00017877
95.25	1.94E-05	0	0.02783667	0.519175	0.1192895	0	1.15E-05	0	0.046932	0	0	0.00279	0.0000032	0	0.00016099
95.75	2.22E-05	0	0.02765583	0.158707	0.11837238	0	1.54E-05	0	0.04708	0	0	0.0027	0.0000024	0	0.00014469
96.25	3.06E-05	0	0.02751583	0.02623267	0.11925788	0	3.85E-06	0	0.046964	0	0	0.00253	0.0000016	0	0.00012593
96.75	3.06E-05	0	0.027895	0.005586	0.11862538	0	2.31E-05	0	0.047224	1.43E-06	0	0.002405	0.0000016	0	9.78E-05
97.25	1.11E-05	0	0.02734667	0.00171	0.11872816	0.0000075	7.69E-06	0	0.047224	0	0	0.002365	0.0000016	0	0.00010222
97.75	5.56E-06	0	0.0278775	0.000608	0.11871234	0	1.92E-05	0	0.046268	0	0	0.00217	0.0000016	0	9.19E-05
98.25	2.22E-05	0	0.02774917	0.00032933	0.11854631	0.0000075	3.85E-06	0	0.041292	0	0	0.002175	0.0000016	0	9.19E-05
98.75	2.22E-05	0	0.02765583	0.00012667	0.11901278	0	1.92E-05	0	0.038156	0	0	0.00201	0	0	7.16E-05
99.25	1.39E-05	0	0.02822167	0.000057	0.11841191	0	7.69E-06	0	0.038308	0	0	0.001935	0.000004	0	7.95E-05
99.75	8.33E-06	0	0.027405	0.000019	0.11845934	0.0000075	1.92E-05	0	0.041692	0	0	0.001885	0.0000024	0	7.56E-05
100.25	2.22E-05	0	0.02797083	0.000019	0.11934484	0	3.85E-06	0	0.061604	0	0	0.00175	0.0000008	0	6.62E-05

100.75	1.39E-05	0	0.02797083	6.33E-06	0.11862538	0.0000075	1.92E-05	0	0.087232	2.86E-06	0	0.001675	0.0000024	0	5.83E-05
101.25	2.22E-05	0	0.02793583	6.33E-06	0.11848306	0.000015	1.15E-05	0	0.069224	0	0	0.001525	0.0000024	0	6.02E-05
101.75	1.39E-05	0	0.02773167	6.33E-06	0.11848306	0.00003	7.69E-06	0	0.059384	0	0	0.001545	0.0000016	0	5.73E-05
102.25	1.39E-05	0	0.02797667	0	0.11811938	0.0000525	7.69E-06	0	0.063196	1.43E-06	0	0.00144	0.0000008	0	5.19E-05
102.75	1.39E-05	0	0.028315	0	0.11831703	0.000135	3.85E-06	0	0.119272	0	0	0.001385	0.0000032	0	4.64E-05
103.25	1.11E-05	0	0.02872917	0	0.11870444	0.00039	1.15E-05	0	0.29906	0	0	0.001315	0.0000016	0	4.44E-05
103.75	1.11E-05	0	0.02786583	6.33E-06	0.11823797	0.00096	1.15E-05	0	0.384616	0	0	0.0013	0	0	4.44E-05
104.25	1.94E-05	0	0.02968	0	0.11958203	0.0020325	0	0	0.210804	1.43E-06	0	0.00119	0.0000008	0	0.00004
104.75	1.94E-05	0	0.03358833	0	0.11879931	0.00588	1.15E-05	0	0.123892	4.29E-06	0	0.00122	0.0000016	0	3.85E-05
105.25	8.33E-06	0	0.04407667	6.33E-06	0.11876769	0.016905	1.54E-05	0	0.10278	2.86E-06	0	0.00112	0.0000024	0	3.65E-05
105.75	1.67E-05	0	0.064225	0	0.11784266	0.0458775	1.15E-05	0	0.0821	1.43E-06	0	0.001095	0.0000016	0	3.46E-05
106.25	1.39E-05	0	0.10704167	0	0.11757384	0.16293	1.54E-05	0	0.071804	4.29E-06	0	0.00102	0.0000024	0	3.70E-05
106.75	1.39E-05	0	0.21326083	0	0.11811938	0.6401175	1.15E-05	0	0.070856	4.29E-06	0	0.00092	0.0000024	0	2.81E-05
107.25	1.67E-05	0	0.40914417	6.33E-06	0.11777941	1.3304775	7.69E-06	0	0.07378	2.86E-06	0	0.000945	0.0000048	0	3.26E-05
107.75	2.78E-06	0	0.6900425	0	0.11838028	0.8572725	1.15E-05	0	0.077392	1.86E-05	0	0.00091	0.0000016	0	2.86E-05
108.25	1.11E-05	0	0.98449167	0	0.11776359	0.3107625	2.31E-05	0	0.073796	1.71E-05	0	0.000825	0.0000016	0	2.91E-05
108.75	1.11E-05	0	1.25092917	6.33E-06	0.11774778	0.226575	7.69E-06	1.11E-05	0.068832	0.00003	0	0.00089	0.0000008	0	2.77E-05
109.25	1.39E-05	0	1.3720525	0	0.11801659	0.217575	3.85E-06	0	0.096652	5.29E-05	0	0.00075	0.0000008	0	2.37E-05
109.75	1.67E-05	0	1.32955667	0	0.11704413	0.2194425	2.69E-05	0	0.231716	0.00012286	0	0.000795	0.0000016	0	2.81E-05
110.25	1.94E-05	0	1.22089333	0	0.11743944	0.2188725	1.54E-05	0	0.369436	0.00017571	0	0.00074	0	0	2.07E-05
110.75	1.67E-05	0	0.97612667	0	0.11766081	0.2189025	2.31E-05	0	0.410932	0.00057714	0	0.00072	0.0000016	0	2.37E-05
111.25	2.78E-06	0	0.7179375	0	0.11860166	0.2214	4.23E-05	1.11E-05	0.413964	0.00194714	0	0.000675	0.0000008	0	2.17E-05
111.75	1.94E-05	0	0.492765	0	0.11845934	0.2228175	3.46E-05	0	0.416736	0.00662714	0	0.000675	0.0000008	0	1.93E-05
112.25	8.33E-06	0	0.33056333	0	0.11848306	0.2241225	3.85E-05	0	0.418584	0.01544429	0	0.000705	0	0	2.42E-05
112.75	8.33E-06	0	0.2160025	0	0.11921834	0.22635	5.77E-05	0	0.422024	0.02461857	0	0.00063	0	0	1.68E-05
113.25	8.33E-06	0	0.15168417	0	0.11927369	0.257175	8.46E-05	0	0.419412	0.02772143	0	0.00058	0.0000016	0	1.73E-05
113.75	1.39E-05	0	0.10742667	0	0.11948716	0.410355	0.00010385	1.11E-05	0.42622	0.02818286	0	0.00054	0.0000008	0	1.63E-05
114.25	8.33E-06	0	0.08190583	0	0.11964528	0.5772975	0.00017308	0	0.47416	0.02839857	0	0.000585	0.0000032	0	1.88E-05
114.75	8.33E-06	0	0.0751625	0	0.12053869	0.62025	0.00025769	0	0.619416	0.02840429	0	0.000545	0.0000008	0	1.78E-05
115.25	0	0	0.07462583	0	0.11989038	0.613455	0.00045	0	0.839616	0.02857857	0	0.000565	0	0	1.28E-05
115.75	2.22E-05	0	0.074725	0	0.12165347	0.56508	0.00077308	0	1.070892	0.02843571	0	0.000455	0.0000016	0	2.02E-05
116.25	2.22E-05	0	0.08288	0	0.12028569	0.485415	0.00139231	2.22E-05	1.131368	0.02858571	0	0.00048	0.0000024	0	1.38E-05
116.75	1.11E-05	0	0.10376333	0	0.1215665	0.37197	0.00318077	1.11E-05	1.084064	0.02985	0	0.000445	0.000004	0	1.38E-05
117.25	8.33E-06	0	0.13939333	0	0.12151906	0.2358375	0.00775385	0	1.096152	0.03773286	0	0.00053	0.0000008	0	1.38E-05
117.75	1.67E-05	0	0.19531167	0	0.12182741	0.12438	0.0215	3.33E-05	1.209764	0.06981143	0	0.000375	0.0000016	0	1.78E-05
118.25	1.11E-05	0	0.26384167	0	0.12170091	0.057495	0.05362692	3.33E-05	1.334064	0.13196286	0	0.00042	0.0000032	0	1.09E-05
118.75	5.56E-06	0	0.34396833	0	0.11946344	0.0261225	0.11386923	0.0001	1.384784	0.19490714	0	0.000405	0	0	1.43E-05
119.25	1.39E-05	0	0.4018525	0	0.11317797	0.0102225	0.1915	0.00021111	1.39414	0.24942571	0	0.00037	0	0	1.33E-05
119.75	1.39E-05	0	0.43183583	0	0.09999034	0.00477	0.28677692	0.00032222	1.360316	0.27404143	0	0.000345	0.0000008	0	1.14E-05
120.25	1.67E-05	0	0.43748833	0	0.08246219	0.002595	0.34606539	0.00274444	1.224648	0.29507143	0	0.000365	0	0	1.33E-05
120.75	5.56E-06	0	0.43142167	0	0.06049072	0.0015225	0.44545769	0.0271	0.794044	0.21997714	0	0.00035	0.0000016	0	1.38E-05
121.25	5.56E-06	0	0.413245	0	0.04242494	0.0008775	0.72438846	0.12346667	0.296888	0.17714429	0	0.00035	0	0	9.38E-06
121.75	2.78E-06	0	0.42929833	0	0.02911081	0.0005775	1.20311923	0.45433333	0.055348	0.22163429	0	0.00034	0	0	1.04E-05
122.25	1.94E-05	0	0.43799	0	0.01959169	0.00042	1.35512692	0.88921111	0.0028	0.33927286	0	0.000325	0.0000016	0	1.09E-05
122.75	8.33E-06	0	0.43317167	0	0.01404941	0.00027	1.17653077	1.20104444	0	0.53705714	0	0.00029	0.0000024	0	1.04E-05
123.25	8.33E-06	0	0.447475	0	0.00991444	0.0002175	1.02850385	1.29035556	0	0.77775429	0	0.000245	0.0000008	0	1.33E-05
123.75	2.78E-06	0	0.43839833	0	0.00730538	0.000135	0.87746923	1.14438889	0	1.08853714	0	0.000305	0.0000008	0	7.41E-06
124.25	5.56E-06	0	0.45178583	0	0.00581109	0.00015	0.64010769	0.92204444	0	1.39902714	0	0.00027	0	0	8.40E-06
124.75	1.67E-05	0	0.46733167	0	0.0044275	0.0000675	0.48145385	0.60418889	0	1.57972857	0	0.000245	0.0000016	0	7.41E-06
125.25	5.56E-06	0	0.47383583	0	0.00335225	0.0001125	0.39242308	0.36475556	0	1.32072714	0	0.00026	0.0000016	0	1.09E-05
125.75	5.56E-06	0	0.4759475	0	0.00288578	0.000045	0.37582692	0.1979	0	0.68030571	0	0.000245	0.0000016	0	8.89E-06
126.25	1.39E-05	0	0.47065083	0	0.00223747	0.0000525	0.49692692	0.08	0	0.40338571	0	0.00029	0.0000008	0	6.42E-06
126.75	1.67E-05	0	0.46807833	0	0.00172356	0.000045	0.87814615	0.03294444	0	0.32647143	0	0.00019	0	0	8.89E-06
127.25	5.56E-06	0	0.45928167	0	0.00157334	0.0000225	1.21403462	0.0143	0	0.35104857	0	0.00027	0	0	7.41E-06
127.75	8.33E-06	0	0.45546083	0	0.00116222	0.0000075	1.36207308	0.00613333	0	0.42761143	0	0.000215	0	0	6.42E-06
128.25	1.11E-05	0	0.42077583	0	0.00103572	0.0000375	1.17365	0.00403333	0	0.65140429	0	0.00026	0	0	8.89E-06
128.75	1.11E-05	0	0.40580167	0	0.00085388	0.0000075	0.81033846	0.00298889	0	0.71961143	0	0.0002	0	0	8.40E-06
129.25	2.78E-06	0	0.36521917	0	0.00070366	0.000015	0.70734231	0.00325556	0	0.42361	0	0.0002	0.0000016	0	6.91E-06
129.75	5.56E-06	0	0.32892417	0	0.00070366	0.00003	0.70646539	0.00286667	0	0.09980429	0	0.00022	0.0000008	0	7.41E-06
130.25	1.11E-05	0	0.30329833	0	0.00052181	0.0000075	0.70581539	0.00302222	0	0.01640571	0	0.000195	0	0	2.96E-06
130.75	2.78E-06	0	0.24871	0	0.00045856	0.0000075	0.76230385	0.00313333	0	0.00148429	0	0.00022	0	0	7.90E-06
131.25	0	0	0.227255	0	0.00041903	0.000015	0.33653846	0.00292222	0	1.86E-05	0	0.000155	0.0000008	0	8.40E-06
131.75	2.78E-06	0	0.18799667	0	0.00031625	0.0000075	0.03639231	0.00288889	0	0	0	0.000215	0.0000016	0	5.93E-06
132.25	0.000025	0	0.17374583	0	0.00029253	0.000015	0.00348462	0.003	0	0	0	0.000155	0	0	6.42E-06
132.75	1.11E-05	0	0.18958917	0	0.00029253	0	0.00138462	0.0029	0	0	0	0.000195	0.0000008	0	6.91E-06
133.25	1.94E-05	0	0.2483425	0	0.00030834	0.0000075	0.00057692	0.00255556	0	0	0	0.000135	0.0000024	0	3.95E-06
133.75	8.33E-06	0	0.3355275	0	0.00013441	0	0.00005	0.00298889	0	0	0	0.000185	0.0000008	0	7.90E-06
134.25	8.33E-06	0	0.45457417	0	0.00017394	0.0000225	1.15E-05	0.00323333	0	0	0	0.00014	0.0000008	0	5.93E-06
134.75	8.33E-06	0	0.62729917	0	0.00023719	0	1.54E-05	0.00266667	0	0	0	0.000165	0.0000008	0	3.95E-06
135.25	1.39E-05	0	0.764505	0	0.00018975	0	1.15E-05	0.00271111	0	0	0	0.000145	0	0	7.90E-06
135.75	5.56E-06	0	0.66894333	0	0.00014231	0	2.31E-05	0.00293333	0	0	0	0.000135	0	0	3.95E-06
136.25	8.33E-06	0	0.56860417	0	0.00017394	0	1.54E-05	0.00248889	0	0	0	0.00015	0.0000016	0	6.42E-06
136.75	0	0	0.36638583	0	0.00011859	0.0000075	1.54E-05	0.00293333	0	0	0	0.00012	0.0000008	0	5.93E-06
137.25	8.33E-06	0	0.17469667	0	8.70E-05	0.0000075	3.85E-06	0.00266667	0	0	0	0.00014	0	0	4.44E-06
137.75	1.11E-05	0	0.08230833	0	0.0001265	0	7.69E-06	0.00283333	0	0	0	0.00015	0	0	6.91E-06
138.25	2.78E-06	0	0.05377167	0	0.00010278	0	7.69E-06	0.00308889	0	0	0	0.0001	0.0000016	0	4.44E-06
138.75	5.56E-06	0	0.0497525	0	8.70E-05	0.0000075	1.92E-05	0.00263333	0	0	0	0.00013	0	0	2.47E-06
139.25	1.39E-05	0	0.04767	0	6.33E-05	0.0000075	1.54E-05	0.00268889	0	0	0	0.000125	0.0000008	0	2.47E-06
139.75	3.06E-05	0	0.04684167	0	7.91E-05	0.0000075	1.92E-05	0.00288889	0	0	0	0.00012	0	0	5.93E-06
140.25	8.33E-06	0	0.04576833	0	8.70E-05	0	2.31E-05	0.00277778	0	0	0	0.000105	0	0	4.44E-06
140.75	1.11E-05	0	0.04557	0	8.70E-05	0	1.15E-05	0.0029	0	0	0	0.000125	0	0	3.46E-06
141.25	2.78E-06	0	0.045045	0	6.33E-05	0.0000075	3.85E-06	0.0029	0	0	0	0.0001	0.0000016	0	4.44E-06
141.75	5.56E-06	0	0.04386667	0	5.53E-05	0	7.69E-06	0.00253333	0	0	0	0.0001	0	0	4.94E-06
142.25	8.33E-06	0	0.04176083	0	4.74E-05	0	2.31E-05	0.00265556	0	0	0	0.000105	0.0000008	0	5.93E-06
142.75	2.78E-06	0	0.04209917	0	7.12E-05	0.0000075	7.69E-06	0.00276667	0	0	0	0.0001	0.0000008	0	2.47E-06
143.25	2.78E-06	0	0.04195917	0	3.16E-05	0	1.54E-05	0.00307778	0	0	0	0.000105	0	0	3.95E-06
143.75	2.78E-06	0	0.04239083	0	4.74E-05	0.0000075	1.92E-05	0.00277778	0	0	0	0.00011	0.0000008	0	3.95E-06
144.25	8.33E-06	0	0.04212833	0	3.16E-05	0	3.08E-05	0.00268889	0	0	0	0.00008	0	0	5.93E-06
144.75	8.33E-06	0	0.0396725	0	7.91E-06	0	2.31E-05	0.00273333	0	0	0	0.000105	0.0000008	0	4.44E-06
145.25	1.11E-05	0	0.0432075	0	3.16E-05	0	0	0.00254444	0	0	0	0.00009	0	0	3.46E-06
145.75	2.78E-06	0	0.04367417	0	3.95E-05	0	3.85E-05	0.00268889	0	0	0	0.000105	0	0	1.98E-06
146.25	1.11E-05	0	0.043015	0	3.16E-05	0	1.92E-05	0.00307778	0	0	0	0.00008	0.0000008	0	4.44E-06
146.75	0	0	0.04753	0	1.58E-05	0	2.31E-05	0.00271111	0	0	0	0.0001	0	0	4.44E-06
147.25	1.39E-05	0	0.0511	0	1.58E-05	0.0000075	1.54E-05	0.00301111	0	0	0	0.00007	0.0000008	0	4.44E-06
147.75	2.78E-06	0	0.054985	0	3.16E-05	0	3.85E-06	0.00292222	0	0	0	0.00007	0.0000008	0	1.98E-06
148.25	2.78E-06	0	0.06146	0	2.37E-05	0	3.85E-06	0.00287778	0	0	0	0.000095	0	0	6.42E-06
148.75	8.33E-06	0	0.06587	0	2.37E-05	0	2.31E-05	0.00268889	0	0	0	0.00007	0.0000008	0	3.46E-06
149.25	0.000025	0	0.07020417	0	1.58E-05	0	3.08E-05	0.00296667	0	0	0	0.00007	0.0000008	0	1.48E-06
149.75	2.78E-06	0	0.08430333	0	0	0	4.23E-05	0.00267778	0	0	0	0.00009	0	0	1.48E-06
150.25	2.78E-06	0	0.10525667	0	7.91E-06	0	1.54E-05	0.00291111	0	0	0	0.000065	0	0	4.44E-06
150.75	8.33E-06	0	0.14604333	0	0	0	1.54E-05	0.00304444	0	0	0	0.00005	0	0	4.94E-06

151.25	8.33E-06	0	0.2466975	0	0	0	7.69E-06	0.00298889	0	0	0	0.000085	0	0	3.95E-06
151.75	5.56E-06	0	0.40356167	0	1.58E-05	0	2.31E-05	0.00284444	0	0	0	0.00006	0.0000008	0	2.47E-06
152.25	2.78E-06	0	0.6283025	0	1.58E-05	0	1.54E-05	0.00271111	0	0	0	0.000065	0	0	2.96E-06
152.75	1.11E-05	0	0.77210583	0	2.37E-05	0	2.31E-05	0.00267778	0	0	0	0.000055	0	0	2.47E-06
153.25	0	0	0.81096167	0	7.91E-06	0	1.54E-05	0.0027	0	0	0	0.00006	0.0000008	0	2.96E-06
153.75	8.33E-06	0	0.6743975	0	0	0	3.46E-05	0.00295556	0	0	0	0.000065	0.0000008	0	4.94E-06
154.25	2.78E-06	0	0.58198	0	0	0	3.85E-05	0.00271111	0	0	0	0.00006	0.0000016	0	2.96E-06
154.75	1.67E-05	0	0.46286333	0	1.58E-05	0	1.15E-05	0.0027	0	0	0	0.00006	0	0	2.96E-06
155.25	2.78E-06	0	0.40585417	0	0	0	3.08E-05	0.00314444	0	0	0	0.00005	0	0	1.48E-06
155.75	2.78E-06	0	0.37333333	0	1.58E-05	0	7.69E-06	0.0026	0	0	0	0.000055	0	0	3.95E-06
156.25	1.11E-05	0	0.33731833	0	7.91E-06	0	1.15E-05	0.00228889	0	0	0	0.00005	0	0	1.98E-06
156.75	5.56E-06	0	0.30081917	0	1.58E-05	0	1.54E-05	0.00233333	0	0	0	0.00005	0	0	3.95E-06
157.25	8.33E-06	0	0.25904667	0	0	0	2.69E-05	0.0029	0	0	0	0.00004	0	0	3.46E-06
157.75	5.56E-06	0	0.21586833	0	7.91E-06	0	2.31E-05	0.00263333	0	0	0	0.000045	0	0	3.95E-06
158.25	2.78E-06	0	0.18244917	0	0	0	1.92E-05	0.00296667	0	0	0	0.00005	0	0	2.96E-06
158.75	5.56E-06	0	0.15337583	0	7.91E-06	0	3.08E-05	0.00305556	0	0	0	0.000035	0	0	1.48E-06
159.25	2.78E-06	0	0.13180417	0	0	0	1.15E-05	0.00307778	0	0	0	0.00004	0	0	9.88E-07
159.75	8.33E-06	0	0.1064	0	0	0	3.46E-05	0.0026	0	0	0	0.00004	0.0000016	0	3.46E-06
160.25	5.56E-06	0	0.08149167	0	1.58E-05	0	3.08E-05	0.00293333	0	0	0	0.000025	0.0000008	0	3.95E-06
160.75	0	0	0.06320417	0	0	0	2.69E-05	0.00253333	0	0	0	0.00005	0	0	2.96E-06
161.25	5.56E-06	0	0.04956	0	0	0	2.69E-05	0.00274444	0	0	0	0.00003	0.0000008	0	3.95E-06
161.75	5.56E-06	0	0.037905	0	1.58E-05	0	7.69E-06	0.00316667	0	0	0	0.000045	0.0000008	0	9.88E-07
162.25	1.39E-05	0	0.03091083	0	7.91E-06	0	1.92E-05	0.00291111	0	0	0	0.00002	0	0	3.46E-06
162.75	0	0	0.02419083	0	0	0	3.85E-05	0.00267778	0	0	0	0.000045	0	0	1.98E-06
163.25	8.33E-06	0	0.01922083	0	7.91E-06	0	1.92E-05	0.00255556	0	0	0	0.000035	0.0000008	0	1.48E-06
163.75	5.56E-06	0	0.01549917	0	7.91E-06	0	3.85E-06	0.00265556	0	0	0	0.00002	0.0000016	0	4.44E-06
164.25	1.11E-05	0	0.01246583	0	0	0	6.15E-05	0.00274444	0	0	0	0.000055	0	0	2.96E-06
164.75	2.78E-06	0	0.00991667	0	0	0	2.31E-05	0.00276667	0	0	0	0.00003	0.0000008	0	2.96E-06
165.25	5.56E-06	0	0.00789833	0	0	0	3.85E-05	0.0032	0	0	0	0.00003	0	0	1.98E-06
165.75	5.56E-06	0	0.0062125	0	0	0	1.54E-05	0.00274444	0	0	0	0.000035	0	0	4.94E-07
166.25	5.56E-06	0	0.0049	0	0	0	1.54E-05	0.00284444	0	0	0	0.000035	0	0	2.47E-06
166.75	1.11E-05	0	0.0037975	0	0	0	3.08E-05	0.00255556	0	0	0	0.00004	0	0	3.46E-06
167.25	2.78E-06	0	0.00295167	0	7.91E-06	0	7.69E-06	0.00327778	0	0	0	0.00004	0.0000008	0	5.43E-06
167.75	5.56E-06	0	0.0022225	0	7.91E-06	0	2.31E-05	0.00253333	0	0	0	0.00003	0.0000008	0	2.47E-06
168.25	5.56E-06	0	0.0021175	0	0	0	3.85E-05	0.00284444	0	0	0	0.000045	0	0	1.48E-06
168.75	1.11E-05	0	0.00158083	0	0	0	3.85E-05	0.00273333	0	0	0	0.000025	0	0	1.98E-06
169.25	5.56E-06	0	0.00131833	0	0	0	4.23E-05	0.00251111	0	0	0	0.00002	0	0	1.98E-06
169.75	5.56E-06	0	0.00119583	0	0	0	3.08E-05	0.00317778	0	0	0	0.00006	0	0	9.88E-07
170.25	2.78E-06	0	0.00093333	0	0	0	2.31E-05	0.00256667	0	0	0	0.000015	0.0000008	0	2.96E-06
170.75	0	0	0.00090417	0	7.91E-06	0	2.69E-05	0.00272222	0	0	0	0.00004	0.0000016	0	2.47E-06
171.25	5.56E-06	0	0.00074667	0	0	0	3.85E-05	0.00294444	0	0	0	0.00002	0	0	1.98E-06
171.75	1.11E-05	0	0.00061833	0	0	0	3.85E-05	0.00282222	0	0	0	0.000045	0.0000008	0	1.98E-06
172.25	2.78E-06	0	0.00060083	0	0	0	3.46E-05	0.00265556	0	0	0	0.00003	0	0	9.88E-07
172.75	2.78E-06	0	0.00039667	0	0	0	4.23E-05	0.00265556	0	0	0	0.000025	0	0	1.48E-06
173.25	8.33E-06	0	0.00046667	0	0	0	2.69E-05	0.00292222	0	0	0	0.00005	0	0	9.88E-07
173.75	5.56E-06	0	0.000385	0	0	0	0.00005	0.00281111	0	0	0	0	0	0	3.95E-06
174.25	5.56E-06	0	0.00037917	0	0	0	4.62E-05	0.00294444	0	0	0	0.00004	0	0	1.48E-06
174.75	0	0	0.00032667	0	7.91E-06	0	2.69E-05	0.00281111	0	0	0	0.000035	0.0000008	0	1.48E-06
175.25	0	0	0.00023333	0	0	0	3.08E-05	0.00305556	0	0	0	0.000025	0	0	9.88E-07
175.75	0	0	0.00028	0	0	0	2.69E-05	0.00278889	0	0	0	0.00003	0	0	1.48E-06
176.25	5.56E-06	0	0.00025083	0	0	0	3.85E-05	0.0029	0	0	0	0.00002	0	0	1.98E-06
176.75	8.33E-06	0	0.00013417	0	0	0	3.08E-05	0.00296667	0	0	0	0.00002	0	0	2.96E-06
177.25	2.78E-06	0	0.00018083	0	0	0	2.31E-05	0.00302222	0	0	0	0.00003	0	0	1.98E-06
177.75	5.56E-06	0	0.00013417	0	0	0	2.69E-05	0.00293333	0	0	0	0.00002	0.0000016	0	1.98E-06
178.25	2.78E-06	0	0.00014583	0	0	0	2.69E-05	0.00286667	0	0	0	0.00002	0	0	4.94E-07
178.75	5.56E-06	0	0.0000875	0	0	0	3.46E-05	0.00285556	0	0	0	0.00002	0	0	1.98E-06
179.25	2.78E-06	0	0.0001225	0	0	0	4.62E-05	0.00278889	0	0	0	0.00002	0.0000008	0	1.48E-06
179.75	2.78E-06	0	9.33E-05	0	0	0	3.08E-05	0.00333333	0	0	0	0.000015	0	0	9.88E-07
180.25	1.39E-05	0	0.0000875	0	0	0	5.38E-05	0.00295556	0	0	0	0.00003	0	0	2.47E-06
180.75	0	0	8.17E-05	0	0	0	3.08E-05	0.00294444	0	0	0	0.00002	0	0	1.98E-06
181.25	2.78E-06	0	9.33E-05	0	0	0	3.85E-05	0.00298889	0	0	0	0.000015	0.0000008	0	2.47E-06
181.75	5.56E-06	0	6.42E-05	0	7.91E-06	0	3.85E-05	0.003	0	0	0	0.000015	0	0	4.94E-07
182.25	5.56E-06	0	4.08E-05	0	0	0	5.38E-05	0.00257778	0	0	0	0.00003	0	0	1.48E-06
182.75	2.78E-06	0	4.08E-05	0	0	0	5.38E-05	0.00282222	0	0	0	0.000015	0	0	9.88E-07
183.25	5.56E-06	0	7.58E-05	0	0	0	2.31E-05	0.00295556	0	0	0	0.000015	0	0	2.96E-06
183.75	5.56E-06	0	5.83E-05	0	0	0	0.00005	0.00293333	0	0	0	0.00002	0	0	1.48E-06
184.25	8.33E-06	0	4.67E-05	0	0	0	4.62E-05	0.00297778	0	0	0	0.00001	0	0	2.96E-06
184.75	5.56E-06	0	0.000035	0	0	0	0.00005	0.00271111	0	0	0	0.000025	0	0	1.98E-06
185.25	2.78E-06	0	0.0000525	0	0	0	5.77E-05	0.0025	0	0	0	0.000015	0	0	0
185.75	0	0	2.92E-05	0	0	0	1.92E-05	0.00285556	0	0	0	0.000015	0.0000008	0	1.48E-06
186.25	0	0	4.08E-05	0	0	0	3.85E-05	0.00298889	0	0	0	0.000025	0	0	1.48E-06
186.75	8.33E-06	0	0.0000175	0	0	0	7.69E-05	0.00252222	0	0	0	0.000005	0	0	1.98E-06
187.25	0	0	0.000035	0	0	0	4.62E-05	0.003	0	0	0	0.000025	0	0	1.48E-06
187.75	5.56E-06	0	0.000035	0	0	0	8.85E-05	0.00268889	0	0	0	0.00001	0.0000008	0	1.48E-06
188.25	0	0	2.33E-05	0	0	0	3.85E-05	0.0024	0	0	0	0.00001	0	0	9.88E-07
188.75	8.33E-06	0	1.17E-05	0	0	0	3.46E-05	0.00264444	0	0	0	0.00003	0.0000008	0	4.94E-07
189.25	2.78E-06	0	2.33E-05	0	7.91E-06	0	6.15E-05	0.00294444	0	0	0	0	0	0	9.88E-07
189.75	0	0	1.17E-05	0	0	0	0.00005	0.00284444	0	0	0	0.000005	0	0	1.98E-06
190.25	2.78E-06	0	1.17E-05	0	0	0	0.00005	0.00291111	0	0	0	0.000025	0	0	9.88E-07
190.75	5.56E-06	0	1.17E-05	0	0	0	3.46E-05	0.00287778	0	0	0	0.000005	0.0000008	0	1.98E-06
191.25	5.56E-06	0	2.33E-05	0	0	0	3.85E-05	0.00287778	0	0	0	0.00001	0	0	9.88E-07
191.75	0	0	5.83E-06	0	0	0	5.38E-05	0.00287778	0	0	0	0.000015	0	0	1.48E-06
192.25	8.33E-06	0	1.17E-05	0	0	0	5.38E-05	0.0031	0	0	0	0	0	0	9.88E-07
192.75	0	0	1.17E-05	0	0	0	6.54E-05	0.00283333	0	0	0	0	0	0	9.88E-07
193.25	2.78E-06	0	5.83E-06	0	0	0	5.77E-05	0.00285556	0	0	0	0.00002	0	0	1.48E-06
193.75	5.56E-06	0	5.83E-06	0	0	0	8.85E-05	0.00278889	0	0	0	0.000005	0	0	4.94E-07
194.25	1.39E-05	0	5.83E-06	0	0	0	7.69E-05	0.00275556	0	0	0	0	0.0000008	0	1.98E-06
194.75	0	0	2.92E-05	0	0	0	7.69E-05	0.00271111	0	0	0	0.00001	0	0	4.94E-07
195.25	2.78E-06	0	0.0000175	0	0	0	5.38E-05	0.0028	0	0	0	0.00001	0	0	1.98E-06
195.75	2.78E-06	0	0	0	0	0	0.00005	0.00271111	0	0	0	0.000005	0.0000008	0	9.88E-07
196.25	2.78E-06	0	1.17E-05	0	0	0	0.00005	0.00266667	0	0	0	0.00001	0	0	1.98E-06
196.75	8.33E-06	0	1.17E-05	0	0	0	6.92E-05	0.00273333	0	0	0	0.000005	0	0	0
197.25	5.56E-06	0	0.0000175	0	0	0	8.85E-05	0.0028	0	0	0	0.000005	0	0	9.88E-07
197.75	0	0	1.17E-05	0	0	0	7.69E-05	0.00285556	0	0	0	0.00001	0	0	1.48E-06
198.25	8.33E-06	0	0	0	0	0	6.92E-05	0.00278889	0	0	0	0	0.0000008	0	1.48E-06
198.75	0	0	0.0000175	0	0	0	5.77E-05	0.00291111	0	0	0	0.000015	0	0	1.48E-06
199.25	0	0	0	0	0	0	6.54E-05	0.00287778	0	0	0	0	0	0	9.88E-07
199.75	2.78E-06	0	0	0	0	0	7.31E-05	0.00266667	0	0	0	0.000005	0	0	1.48E-06
200.25	8.33E-06	0	0	0	0	0	9.62E-05	0.00277778	0	0	0	0.00001	0	0	1.48E-06
200.75	2.78E-06	0	0	0	0	0	0.00005	0.00287778	0	0	0	0	0	0	1.98E-06
201.25	0	0	5.83E-06	0	0	0	6.92E-05	0.00296667	0	0	0	0.00001	0	0	4.94E-07

201.75	0	0	0	0	0	0	5.77E-05	0.00283333	0	0	0	0	0	0	9.88E-07
202.25	2.78E-06	0	1.17E-05	0	0	0	8.46E-05	0.00287778	0	0	0	0.00001	0	0	4.94E-07
202.75	2.78E-06	0	5.83E-06	0	0	0	6.15E-05	0.00264444	0	0	0	0.000005	0.0000032	0	1.48E-06
203.25	0	0	1.17E-05	0	0	0	6.92E-05	0.00294444	0	0	0	0	0	0	4.94E-07
203.75	5.56E-06	0	0	0	0	0	9.23E-05	0.00261111	0	0	0	0.00001	0	0	9.88E-07
204.25	1.11E-05	0	0	0	0	0	0.0001	0.00263333	0	0	0	0.00001	0	0	1.48E-06
204.75	0	0	1.17E-05	0	0	0	7.69E-05	0.00303333	0	0	0	0	0.0000008	0	9.88E-07
205.25	5.56E-06	0	5.83E-06	0	0	0	8.08E-05	0.00282222	0	0	0	0	0	0	4.94E-07
205.75	2.78E-06	0	0	0	0	0	9.62E-05	0.00277778	0	0	0	0.000015	0.0000008	0	3.46E-06
206.25	2.78E-06	0	0	0	0	0	7.69E-05	0.00286667	0	0	0	0	0	0	9.88E-07
206.75	2.78E-06	0	5.83E-06	0	0	0	0.0001	0.00272222	0	0	0	0.000005	0	0	4.94E-07
207.25	0	0	5.83E-06	0	0	0	0.00010769	0.00286667	0	0	0	0.00001	0	0	9.88E-07
207.75	8.33E-06	0	0	0	0	0	0.00011154	0.00294444	0	0	0	0.000005	0	0	9.88E-07
208.25	2.78E-06	0	0	0	0	0	0.00011923	0.00288889	0	0	0	0	0	0	9.88E-07
208.75	5.56E-06	0	0	0	0	0	0.00012308	0.00301111	0	0	0	0.00001	0.0000008	0	4.94E-07
209.25	2.78E-06	0	0	0	0	0	9.23E-05	0.00292222	0	0	0	0.00001	0	0	9.88E-07
209.75	0	0	5.83E-06	0	0	0	9.23E-05	0.00321111	0	0	0	0	0	0	4.94E-07
210.25	2.78E-06	0	0	0	0	0	0.00012692	0.00312222	0	0	0	0.000005	0	0	1.48E-06
210.75	2.78E-06	0	0	0	0	0	0.00010385	0.00321111	0	0	0	0.000005	0	0	4.94E-07
211.25	2.78E-06	0	0	0	0	0	0.00010385	0.00366667	0	0	0	0.000005	0.0000016	0	9.88E-07
211.75	0	0	0	0	0	0	0.00010769	0.00337778	0	0	0	0.00001	0.0000008	0	0
212.25	2.78E-06	0	1.17E-05	0	0	0	0.00011923	0.00345556	0	0	0	0.000005	0.0000008	0	4.94E-07
212.75	5.56E-06	0	1.17E-05	0	0	0	0.00011539	0.00451111	0	0	0	0	0	0	4.94E-07
213.25	5.56E-06	0	0	0	0	0	0.00013462	0.00612222	0	0	0	0.000005	0	0	9.88E-07
213.75	2.78E-06	0	5.83E-06	0	0	0	0.00012308	0.0079	0	0	0	0.000005	0.0000008	0	4.94E-07
214.25	0	0	5.83E-06	0	0	0	0.00014615	0.0119	0	0	0	0.00001	0	0	1.48E-06
214.75	0	0	0	0	0	0	0.00013846	0.01577778	0	0	0	0.000005	0	0	0
215.25	0	0	0	0	0	0	0.00014231	0.02012222	0	0	0	0.000005	0	0	4.94E-07
215.75	5.56E-06	0	0	0	0	0	0.00011923	0.02322222	0	0	0	0.000005	0	0	4.94E-07
216.25	2.78E-06	0	0	0	0	0	0.00013846	0.02697778	0	0	0	0	0	0	1.48E-06
216.75	5.56E-06	0	0	0	0	0	0.00015769	0.02664444	0	0	0	0.00001	0.0000008	0	0
217.25	2.78E-06	0	0	0	0	0	0.00016154	0.02756667	0	0	0	0.00001	0.0000008	0	4.94E-07
217.75	0	0	0	0	0	0	0.00017692	0.02543333	0	0	0	0	0	0	0
218.25	0	0	5.83E-06	0	0	0	0.00015769	0.0234	0	0	0	0.00001	0.0000016	0	0
218.75	2.78E-06	0	0	0	0	0	0.00016923	0.02462222	0	0	0	0	0	0	9.88E-07
219.25	2.78E-06	0	0	0	0	0	0.00012308	0.02464444	0	0	0	0.000005	0	0	0
219.75	0	0	0	0	0	0	0.00021539	0.02607778	0	0	0	0.00001	0	0	0
220.25	0	0	5.83E-06	0	0	0	0.00016923	0.02581111	0	0	0	0.000005	0	0	1.48E-06
220.75	2.78E-06	0	0	0	0	0	0.00013846	0.02551111	0	0	0	0.000005	0	0	0
221.25	0	0	5.83E-06	0	0	0	0.00019231	0.02523333	0	0	0	0.000005	0	0	4.94E-07
221.75	2.78E-06	0	0	0	0	0	0.00026923	0.0253	0	0	0	0	0	0	4.94E-07
222.25	5.56E-06	0	0	0	0	0	0.00023077	0.02604444	0	0	0	0.00001	0	0	0
222.75	0	0	5.83E-06	0	0	0	0.00021923	0.02586667	0	0	0	0.00001	0	0	0
223.25	0	0	0	0	0	0	0.00019615	0.02534444	0	0	0	0	0	0	0
223.75	2.78E-06	0	0	0	0	0	0.00027692	0.02513333	0	0	0	0.000005	0	0	1.48E-06
224.25	5.56E-06	0	0	0	0	0	0.00021923	0.02627778	0	0	0	0.00001	0	0	4.94E-07
224.75	0	0	0	0	0	0	0.00026154	0.0262	0	0	0	0	0	0	0
225.25	0	0	0	0	0	0	0.00026539	0.02636667	0	0	0	0.000005	0	0	4.94E-07
225.75	0	0	0	0	0	0	0.0003	0.02776667	0	0	0	0.000015	0	0	0
226.25	2.78E-06	0	0	0	0	0	0.00024231	0.03224444	0	0	0	0	0	0	0
226.75	0	0	0	0	0	0	0.00026923	0.04601111	0	0	0	0	0	0	4.94E-07
227.25	1.11E-05	0	5.83E-06	0	0	0	0.00025769	0.06252222	0	0	0	0.000005	0	0	9.88E-07
227.75	8.33E-06	0	0	0	0	0	0.00036154	0.09938889	0	0	0	0.000015	0	0	0
228.25	2.78E-06	0	0	0	0	0	0.00038077	0.14702222	0	0	0	0	0	0	4.94E-07
228.75	0	0	0	0	0	0	0.00036539	0.19507778	0	0	0	0	0	0	9.88E-07
229.25	8.33E-06	0	0	0	0	0	0.00040769	0.27616667	0	0	0	0.000015	0	0	0
229.75	2.78E-06	0	0	0	0	0	0.00032308	0.37286667	0	0	0	0	0	0	4.94E-07
230.25	0	0	0	0	0	0	0.00044615	0.36592222	0	0	0	0.000005	0.0000008	0	4.94E-07
230.75	2.78E-06	0	5.83E-06	0	0	0	0.00040769	0.50485556	0	0	0	0.000005	0	0	0
231.25	0	0	0	0	0	0	0.00051539	0.57432222	0	0	0	0.00001	0.0000008	0	0
231.75	0	0	0	0	0	0	0.00045385	0.58305556	0	0	0	0	0	0	0
232.25	2.78E-06	0	0	0	0	0	0.00051539	0.54798889	0	0	0	0.000005	0	0	9.88E-07
232.75	0	0	5.83E-06	0	0	0	0.00048077	0.5189	0	0	0	0.000015	0	0	4.94E-07
233.25	0	0	0	0	0	0	0.00060385	0.41462222	0	0	0	0	0	0	4.94E-07
233.75	2.78E-06	0	0	0	0	0	0.00060769	0.38512222	0	0	0	0.000005	0	0	4.94E-07
234.25	0	0	0	0	0	0	0.00066539	0.34176667	0	0	0	0.000005	0	0	4.94E-07
234.75	0	0	0	0	0	0	0.00065385	0.30154444	0	0	0	0.000005	0	0	4.94E-07
235.25	2.78E-06	0	0	0	0	0	0.00080385	0.28291111	0	0	0	0.000005	0	0	0
235.75	0	0	0	0	0	0	0.00078077	0.22984444	0	0	0	0.000005	0	0	0
236.25	2.78E-06	0	0	0	0	0	0.00075769	0.20277778	0	0	0	0.000005	0	0	0
236.75	2.78E-06	0	5.83E-06	0	0	0	0.00094615	0.19782222	0	0	0	0.000005	0	0	0
237.25	0	0	0	0	0	0	0.00088846	0.18334444	0	0	0	0	0	0	4.94E-07
237.75	2.78E-06	0	0	0	0	0	0.0011	0.17417778	0	0	0	0.000005	0	0	0
238.25	0	0	0	0	0	0	0.0011	0.16178889	0	0	0	0.00001	0	0	9.88E-07
238.75	2.78E-06	0	0	0	0	0	0.00118846	0.14623333	0	0	0	0	0	0	0
239.25	2.78E-06	0	0	0	0	0	0.00129231	0.12927778	0	0	0	0	0	0	4.94E-07
239.75	2.78E-06	0	0	0	0	0	0.00145	0.11726667	0	0	0	0.000005	0	0	4.94E-07
240.25	2.78E-06	0	0	0	0	0	0.00160769	0.11091111	0	0	0	0.000005	0	0	4.94E-07
240.75	2.78E-06	0	0	0	0	0	0.00178846	0.09751111	0	0	0	0.000005	0	0	0
241.25	2.78E-06	0	0	0	0	0	0.00204615	0.09455556	0	0	0	0.000005	0	0	0
241.75	0	0	0	0	0	0	0.00214615	0.08196667	0	0	0	0.000005	0	0	4.94E-07
242.25	8.33E-06	0	0	0	0	0	0.00263846	0.06958889	0	0	0	0	0	0	0
242.75	2.78E-06	0	0	0	0	0	0.00288462	0.05152222	0	0	0	0.000005	0	0	4.94E-07
243.25	0	0	0	0	0	0	0.00353077	0.03696667	0	0	0	0	0	0	4.94E-07
243.75	0	0	0	0	0	0	0.00450385	0.02791111	0	0	0	0.00001	0.0000008	0	0
244.25	2.78E-06	0	0	0	0	0	0.00750769	0.02091111	0	0	0	0	0	0	4.94E-07
244.75	2.78E-06	0	0	0	0	0	0.01234615	0.01302222	0	0	0	0.000005	0	0	4.94E-07
245.25	2.78E-06	0	0	0	0	0	0.0238	0.00981111	0	0	0	0	0	0	0
245.75	0	0	0	0	0	0	0.06418462	0.00826667	0	0	0	0.000005	0	0	0
246.25	0	0	0	0	0	0	0.11598077	0.00678889	0	0	0	0.00001	0	0	0
246.75	2.78E-06	0	0	0	0	0	0.21636154	0.00628889	0	0	0	0	0.0000008	0	4.94E-07
247.25	0	0	5.83E-06	0	0	0	0.29289231	0.00566667	0	0	0	0	0	0	4.94E-07
247.75	0	0	0	0	0	0	0.33734615	0.00507778	0	0	0	0.000005	0	0	0
248.25	2.78E-06	0	0	0	0	0	0.25947308	0.00514444	0	0	0	0	0	0	9.88E-07
248.75	0	0	0	0	0	0	0.21463077	0.00535556	0	0	0	0.000005	0.0000008	0	9.88E-07
249.25	2.78E-06	0	0	0	0	0	0.1193	0.00544444	0	0	0	0	0	0	9.88E-07
249.75	0	0	0	0	0	0	0.0766	0.00528889	0	0	0	0.000005	0	0	0
250.25	0	0	0	0	0	0	0.03355385	0.00554444	0	0	0	0	0	0	0
250.75	2.78E-06	0	0	0	0	0	0.00928846	0.00525556	0	0	0	0	0	0	0
251.25	0	0	0	0	0	0	0.00187692	0.00526667	0	0	0	0.000005	0	0	0
251.75	0	0	5.83E-06	0	0	0	0.00030385	0.00536667	0	0	0	0	0	0	0

252.25	0	0	0	0	0	0	0.00161923	0.00541111	0	0	0	0	0	0	0
252.75	0	0	0	0	0	0	0.00011923	0.00533333	0	0	0	0	0	0	4.94E-07
253.25	2.78E-06	0	5.83E-06	0	0	0	4.62E-05	0.00535556	0	0	0	0	0	0	0
253.75	0	0	0	0	0	0	7.69E-06	0.00517778	0	0	0	0	0	0	0
254.25	2.78E-06	0	0	0	0	0	3.08E-05	0.00531111	0	0	0	0.000005	0	0	1.48E-06
254.75	2.78E-06	0	0	0	0	0	1.15E-05	0.00552222	0	0	0	0	0	0	0
255.25	2.78E-06	0	0	0	0	0	3.85E-06	0.00553333	0	0	0	0	0	0	0
255.75	0	0	0	0	0	0	7.69E-06	0.00502222	0	0	0	0	0	0	4.94E-07
256.25	2.78E-06	0	0	0	0	0	1.15E-05	0.00494444	0	0	0	0	0	0	4.94E-07
256.75	5.56E-06	0	0	0	0	0	0	0.0051	0	0	0	0.000005	0	0	0
257.25	0	0	0	0	0	0	3.85E-06	0.00534444	0	0	0	0	0	0	0
257.75	5.56E-06	0	0	0	0	0	0	0.00544444	0	0	0	0	0	0	9.88E-07
258.25	2.78E-06	0	0	0	0	0	0	0.00516667	0	0	0	0	0	0	0
258.75	0	0	0	0	0	0	0	0.00544444	0	0	0	0	0	0	0
259.25	0	0	0	0	0	0	3.85E-06	0.00527778	0	0	0	0.000005	0.0000008	0	4.94E-07
259.75	2.78E-06	0	0	0	0	0	0	0.00527778	0	0	0	0	0	0	9.88E-07
